# Predictive capacity of trauma indices for hospitalization and intensive care unit admission

**DOI:** 10.1590/1980-220X-REEUSP-2025-0092en

**Published:** 2025-09-05

**Authors:** Lillian Caroline Fernandes, Daniel Bueno Damasceno, Fernanda Naves de Oliveira Lima, Lilia de Souza Nogueira, Ramon Antonio Oliveira, Filipe Utuari de Andrade Coelho, Regina Marcia Cardoso de Sousa

**Affiliations:** 1 Universidade de São Paulo, Escola de Enfermagem, Departamento de Enfermagem Médico-Cirúrgica, São Paulo, SP, Brazil.; 2 Hospital Israelita Albert Einstein, Faculdade de Ciências da Saúde Albert Einstein, São Paulo, SP, Brazil.

**Keywords:** External Causes, Trauma Severity Indices, Intensive Care Units, Nursing

## Abstract

**Objective:**

To compare the performance of trauma severity indices (ISS, NISS, REMS, mREMS) in predicting hospital and Intensive Care Unit (ICU) admission outcomes.

**Method:**

Retrospective cohort study carried out with patients treated at the Emergency Room of a private hospital from January 2020 to January 2022. Medical records of adults with blunt, penetrating, or mixed trauma admitted up to 24 hours after the trauma were analyzed. Severity indices were used to predict hospital and ICU admission.

**Results:**

The sample consisted of 151 patients. The ISS and NISS showed good discrimination capacity for hospital and ICU admission (AUC/ROC from 0.84 to 0.85). The REMS and mREMS indices showed insufficient performance (AUC/ROC from 0.62 to 0.67).

**Conclusion:**

The ISS and NISS indices were effective in predicting hospital and ICU admission and can be used in clinical practice; however, the use of REMS and mREMS cannot be recommended due to insufficient performance for these outcomes.

## INTRODUCTION

Trauma is caused by the action of external agents resulting in injuries to the individual^(1)^ and has great global relevance due to its high incidence, mortality, hospital costs and its potential to generate temporary or permanent disabilities. Data from the Brazilian Public Health System’s Information Technology Department (*DATASUS*) show that external causes were responsible for 1,557,896 hospitalizations in 2024, with a mortality rate of 2.10% and a total cost of R$2,280,591,137.83 to the Brazilian Public Health System (*SUS*). In this context, the population aged 20–59 stands out, accounting for around 60.63% of cases^(2)^.

Based on this data, it is possible to infer the importance of providing adequate care to this group of individuals. In trauma, the quality of patient care is directly related to the efficiency of the professionals who work in their care, from pre-hospital care to hospital discharge, including the rehabilitation and reintegration of this individual into society^(3,4)^. To guarantee this specialized assistance, both public and private institutions must have adequate infrastructure and trained professionals. For this purpose, Ordinance No. 1,336 was established to organize trauma centers in Brazil, reinforcing the need for a structured and efficient system^(5)^.

When implementing this system, so that immediate interventions and screenings are effective and to ensure adequate care planning, it is essential that everyone involved in the care is aware of the severity of each patient’s clinical condition^(6)^. To this end, trauma centers often use trauma severity indices to assist in care and management processes. These metrics provide an objective measure of injury severity and estimate outcomes, allowing for more accurate and assertive action planning^(7,8)^.

Trauma severity indices are systems that use anatomical (anatomical indices), physiological (physiological indices) changes, or both for scoring. Among the main physiological indices are the *Rapid Emergency Medicine Score* (REMS)^(9)^ and the *Modified Rapid Emergency Medicine Score* (mREMS)^(10)^ and, among the anatomical ones, the *Injury Severity Score* (ISS)^(11)^ and the *New Injury Severity Score* (NISS)^(12)^ have been highlighted.

Hospital admission to the Intensive Care Unit (ICU) and death have stood out as outcomes of interest for trauma patients^(13,14)^. However, no studies were found that evaluated the predictive capacity of trauma severity indices for hospital admission, and few studies evaluated indices for ICU admission^(15,16)^, and research analyzing the prognostic capacity for death was frequent^(3,6,16,17)^. The occurrence of hospitalization and ICU admission has aroused interest because they are associated with complex procedures, hospital costs, and late complications^(13,14)^ after trauma.

The application of severity indices, associated with the detailed recording of traumatic events, has several clinical and scientific uses, contributing to improving patient care. Although there are several indices available, it is essential to identify those that are most accurate and reliable at different stages of care. Furthermore, there is a lack of studies aimed at evaluating the predictive capacity of severity indices in relation to ICU and hospital admissions. Given this scenario, this study aims to compare the performance of trauma indices (ISS, NISS, REMS and mREMS) in predicting these outcomes.

## METHOD

### Design of Study

Retrospective cohort study, written in accordance with the recommendations of the *STrengthening the Reporting of OBservational studies in Epidemiology* (STROBE)^(18)^, carried out through the analysis of electronic medical records of trauma patients admitted to the Emergency Room of a private hospital in the city of São Paulo. Sociodemographic information was collected, as well as data related to trauma, pre-hospital care, and hospital care.

### Study Local

The study was carried out in a private hospital institution, located in the southern region of the city of São Paulo, characterized as extra-large, with more than 700 active beds. The Adult Emergency Room at this hospital has 34 stretchers and 31 armchairs for the care and assistance of individuals in urgent or emergency situations who, depending on their clinical conditions, are referred to specific treatment units. The institution is considered a type II trauma center, a reference for care of trauma patients who have access to the supplementary health system or socioeconomic conditions to cover the costs of the necessary assistance.

The hospital has a trauma care team consisting of a nurse, a general surgeon, and an orthopedist, which is activated by the Emergency Room team based on physiological and anatomical criteria and the mechanism of injury.

The hospital has adult ICU units spread across two floors, totaling 54 active beds. Each bed is equipped with a continuous multiparametric monitor and structure for ventilatory support, administration of vasoactive drugs, sedation, and performance of highly complex procedures. These units have a general care profile, and trauma patients are admitted to any of the available beds.

### Sample and Selection Criteria

The sample for this study consisted of trauma patients admitted to the Adult Emergency Room between January 1, 2020 and January 1, 2022, according to information provided by the Medical and Statistical Archives Service of the local hospital institution of this study.

Study participants met the following inclusion criteria: individuals with blunt, penetrating, or mixed trauma; age ≥18 years; admitted to the institution within 24 hours of the traumatic event. Individuals admitted in cardiorespiratory arrest, patients with burns, and those transferred from other institutions were excluded, since treatments prior to admission to the study site may alter the physiological variables used to calculate severity indices.

### Data Collection

Data collection was carried out after the research project was approved by the Research Ethics Committee and the Emergency Room Coordination of the institution. After institutional authorizations, the Medical and Statistical Archive Service was asked to send a list containing the electronic addresses of all those treated at the Emergency Room as a result of accidents or violence during the study period.

In a list provided by this service, the medical record numbers and email addresses of patients admitted due to external causes to the emergency sector during the period established in the research were identified in a Microsoft Excel spreadsheet with restricted access via the internal network and corporate email.

Through this list, eligible subjects were contacted via institutional email, with the aim of inviting them to participate in the research. The email sent contained, as an attachment, the data collection instrument and the link to access the Free and Informed Consent Form (FICF). In the absence of a response to this approach, new attempts of contact were made and up to five emails with this content were sent.

Data were collected between July 2023 and July 2024, through the consultation of the electronic medical record, after signing of the FICF. The dependent variables were hospital admission and ICU admission. The independent variables were the severity indices: REMS, mREMS, ISS, and NISS.

REMS is a physiological indicator of severity consisting of the Glasgow Coma Scale (GCS), heart rate (HR), mean arterial pressure (MAP), respiratory rate (RR), oxygen saturation (SatO_2_), and age. According to the values observed upon admission to the emergency service, these variables receive a score from zero to four points, except for age, which varies from zero to six^(9)^. REMS was developed to be applied to non-surgical patients admitted to the emergency room; however, its applicability to trauma patients has been demonstrated^(19)^.

With the aim of improving its predictive capacity for outcomes in the trauma population, mREMS was developed in 2017^(10)^. In this instrument, the weighting of the variables age and GCS was changed and MAP was replaced by systolic blood pressure (SBP). For mREMS, the GCS score ranges from 0 to 6 and the other parameters score from 0 to 4^(10,12)^. The sum of the scores obtained in the REMS and mREMS variables results in index scores ranging from zero to 26, with higher scores indicating greater severity.

The calculation of ISS^(11)^ and NISS^(12)^ follows the guidelines of *Abbreviated Injury Scale* (AIS)^(20)^, a manual that presents a list with a description of numerous injuries, used to identify each patient’s injury and its severity. This list provides, for each injury description, an identifier consisting of seven numbers, the last digit being the value referring to the severity of the injury. The ISS and NISS use in their calculation the severity of injuries established by the AIS and, through specific calculation methods, estimate the overall severity of a patient^(12)^. The ISS and NISS scores range from 1 to 75, with an AIS of 6 automatically assigning an ISS of 75, suggesting a fatal condition.

To characterize the sample studied, sociodemographic variables (sex and age) and information related to the traumatic event (trauma mechanism; external cause; pre-hospital care; type of support; and pre-hospital transport) were collected. From hospital admission, clinical parameters were analyzed and, from the hospital stay period, surgical approaches, destination after care in the Emergency Room, and length of hospital stay were considered.

Data were collected using a data collection instrument, and an injury registration form was created to calculate anatomical severity indices.

### Data Analysis and Treatment

All information collected was stored in a computerized database, protected with a password, to guarantee information security. Furthermore, all identifiable data were encrypted and anonymized. The software Jamovi® version 2.3.26.0 was used for statistical analyses.

The external causes identified in the participants’ medical records in this study were categorized according to Chapter XX – External causes of morbidity and mortality (V01-Y98) of the International Classification of Diseases, 10th Revision (ICD 10)^(21)^. In cases where the trauma could not be specified in this chapter, ICD code T14.9 was used, corresponding to “unspecified trauma”, belonging to Chapter XIX of this classification.

REMS and mREMS were calculated from physiological data recorded during the initial care of patients admitted to the Emergency Room. The scores of these indices were established by adding the scores attributed to the physiological variables that make up the instruments^(9,10)^.

To calculate the ISS, the squares of the highest AIS scores of the three body regions most affected by the trauma were summed^(11)^. To determine the ISS, the most severe injuries were identified in each of the affected regions and the three highest AIS scores in different regions were selected. The calculation was performed by adding the square of these three values: ISS = (AIS1)^2^ + (AIS2)^2^ + (AIS3)^2^. To establish the NISS value, the squares of the three highest AIS scores were added, regardless of the affected body region^(12)^.

To establish the ISS and NISS values, all patient lesions were considered. It should be noted that the anatomical indices were calculated in duplicate and independently. In case of disagreement between the evaluators, a third member, with extensive experience in injury coding, was consulted. At the end of this stage, all coding and calculations were reviewed and validated by this third member.

The Shapiro-Wilk test was performed to check the distribution of the data and, since they did not present a normal distribution, Mann-Whitney test was applied in comparisons of groups of patients admitted or not to hospital units and the ICU*.* To assess the predictive capacity of trauma indices, Receiver Operating Characteristic (ROC) curves were constructed for the study outcomes of interest (hospital admission and ICU admission).

The 95% Confidence Interval of the area under the ROC curve (AUC/ROC) was used to infer statistically significant differences between AUC/ROC values^(22)^. In addition, Youden index was performed to select the cutoff point and obtain measures of sensitivity, specificity, positive predictive value (PPV), negative predictive value (NPV), and accuracy.

The categorization of the AUC/ROC metric values followed the format proposed in the scientific literature: ≤0.6 indicating random behavior; >0.6 and ≤0.7 insufficient discrimination ability; >0.7 and ≤0.8 was considered acceptable; >0.8 and ≤0.9 good discrimination ability; and >0.9, excellent^(23)^. The significance level adopted in all analyses was 5%.

### Ethical Aspects

The study complied with the Guidelines and Regulatory Standards for Research involving human beings, emanating from Resolution No. 466/12 of the Brazilian Health Council^(24)^. The project was submitted and approved by the Research Ethics Committee (CEP), via the platform Brazil (opinion no. 6,107,403 – CAAE 68381923.4.0000.0071).

The FICF was applied electronically. Initially, researchers contacted potential study participants via email to invite them to participate. Along with the invitation text, the data collection files in PDF format and the link to access the TCLE, made available via REDCap® (Vanderbilt, Nashville, TN), were presented.

After signing, a copy of the documents was sent to the patient and/or family member for possible future consultations. On this occasion, the researchers provided guidance on the importance of archiving the copy and informed participants that they could withdraw their consent at any stage of the study. If this were to occur, an email would be sent confirming the removal of their information from the study. The FICF was preferably applied to the patient and, only in cases where understanding of the term was compromised or in the event of death, it was applied to the family member.

## RESULTS

During the study period, 264 patients were treated at the Emergency Room due to accidents or violence. After applying the eligibility criteria, 42 patients (15.90%) were excluded, 31 due to age under 18 years (11.74%), nine due to having been transferred from other institutions (3.40%) and two due to a specific external cause (burn) (0.75%). Thus, 222 trauma patients met the eligibility criteria (84.09%).

For these patients, the FICF was sent via email, requesting consent for the use of their hospital records in the research. However, 71 patients (31.98%) did not respond after five contact attempts or refused to participate. Thus, the sample consisted of 151 participants. Among them, 75 patients (49.66%) were required to be hospitalized and 14 (9.27%) were admitted to the ICU.

The average age of the patients was 52.40 years, the median was 47 years, with minimum and maximum values of 19 and 100 years, respectively. The highest frequency was of men (55%), and blunt trauma was the main mechanism of injury (95.40%). Among external causes, falls and accidents involving injured cyclists had the highest incidence (43.71% and 16.56%, respectively).

Regarding pre-hospital care, 57% of participants did not receive this type of care; the others received, more frequently, basic care (37.10%) by ground teams (41.70%), as shown in Table 1.

**Table 1:** Data related to trauma, hospital care, and trauma severity indices of research participants (n = 151) – São Paulo, 2020–2022.

Trauma-related variables	
Trauma mechanism – n (%)	
Blunt	144 (95.40)
Penetrating	7 (4.60)
External cause / ICD10 code – n (%)	
Falls / W00-W19	66 (43.71)
Pedal cyclist injured / V10-V19	25 (16.56)
Motorcycle rider injured in transport accident / V20-V29	17 (11.26)
Car occupant injured in transport accident / V40-V49	15 (9.93)
Pedestrian injured in transport accident / V01-V09	6 (3.97)
Exposure to inanimate mechanical forces / W20-W49	6 (3.97)
Other land transport accidents / V80-V89	4 (2.65)
Other traumas* / V30-V39; V90-V94; X85-Y09; X58-X59; X60-X84	5 (3.31)
Unspecified injury / T14.9	7 (4.64)
Type of pre-hospital support – n (%)	
Basic	56 (37.10)
Intermediate	5 (3.30)
Advanced	4 (2.60)
No support	86 (57.00)
Type of prehospital transport – n (%)	
Air	2 (1.30)
Terrestrial	63 (41.70)
Not applicable	86 (57.00)
Vital signs on admission to the Emergency Room – median (Q1–Q3)	
Heart rate, beats per minute	77.00 (69.00–89.00)
Systolic Blood Pressure, millimeters of mercury	123.00 (105–142)
Diastolic Blood Pressure, millimeters of mercury	85.00 (74.50–99.50)
Mean Arterial Pressure, millimeters of mercury	97.70 (88.20–109.00)
Glasgow Coma Scale	15.00 (15.00–15.00)
Peripheral oxygen saturation, %	98.00 (96.00–99.00)
Axillary temperature, degrees Celsius	36.1 (36.00–36.50)
**Variáveis relacionadas ao atendimento hospitalar**	
Destination after emergency room care – n (%)	
High	76 (50.33)
Operating Suite	25 (16.56)
Observation in the Emergency Room	1 (0.66)
Inpatient Unit	31 (20.53)
Semi-Intensive Unit	4 (2.65)
Intensive Care Unit	14 (9.27)
Surgical approach – n (%)	
Yes	51 (33.80)
No	100 (66.20)
Hospital transfer – n (%)	
Yes	13 (8.60)
No	138 (91.40)
Outcome – n (%)	
Survivor	150 (99.30)
Death	1 (0.70)
Length of hospital stay – in days	
Median (Q1–Q3)	1.00 (0.00–3.00)
Trauma severity index, median (Q1–Q3)	
*Injury Severity Score*(ISS)	3.50 (1.00–8.00)
*New Injury Severity Score*(NISS)	3.50 (1.00–9.00)
*Rapid Emergency Medicine Score*(REMS)	2.00 (0.00–5.00)
*Modified Rapid Emergency Medicine Score*(mREMS)	1.00 (0.00–3.00)

Data expressed as number and percentage (%) or as median and quartile 1 (25%), Q1 and quartile 3 (75%), Q3. (*) Other traumas: occupant of three-wheeled motor vehicle / V30-V39 (n = 1); water transport accidents / V90-V94 (n = 1); accidental exposure to other or unspecified factors / X58-X59 (n = 1); intentional self-harm / X60-X84 (n = 1); assault/ X85-Y09 (n = 1).

It was found that 2.71% of participants presented some type of change in clinical parameters upon admission to the Emergency Room. Surgical approach was indicated for 51 patients (33.80%), and 13 study participants (8.60%) were transferred to another hospital. The median length of hospital stay was 1.00 days (Q1: 0.00 – Q3: 3.00) and only one hospital death was recorded. The median values of the severity indices are described in Table 1.

As shown in Table 2, patients who required hospitalization had higher values in the trauma severity indices compared to those who were not hospitalized. A statistically significant difference was observed between the groups with and without hospital admission in relation to the indices analyzed. When comparing patients admitted or not to the ICU, higher values were observed among those admitted to this unit. There was a statistically significant difference in ISS values (p < 0.001), NISS (p < 0.001), REMS (p = 0.027); however, no statistically significant difference was observed in the mREMS values (p = 0.103).

**Table 2:** Comparison of severity indices of trauma patients with and without hospital admission and ICU admission – Sao Paulo, 2020–2022.

Trauma severity index, median (Q1–Q3)	Hospital admission		p^M^
	Yes (n = 75)	No (n = 76)	
*Injury Severity Score*(ISS)	6.00 (4.00–13.00)	1.00 (1.00–2.00)	<0.001
*New Injury Severity Score*(NISS)	6.00 (4.00–15.00)	1.00 (1.00–2.00)	<0.001
*Rapid Emergency Medicine Score*(REMS)	2.00 (0.00–6.00)	0.00 (0.00–4.00)	0.007
*Modified Rapid Emergency Medicine Score*(mREMS)	2.00 (1.00–4.00)	0.00 (0.00–3.00)	0.002
**Trauma severity index, median (Q1–Q3)**	**ICU admission**		**p^M^**
	**Yes (n = 14)**	**No (n = 137)**	
*Injury Severity Score*(ISS)	10.00 (8.25–13.80)	2.00 (1.00–5.00)	<0.001
*New Injury Severity Score*(NISS)	14.00 (9.00–17.00)	2.00 (1.00–6.00)	<0.001
*Rapid Emergency Medicine Score*(REMS)	5.50 (1.00–7.00)	2.00 (1.00–4.00)	0.027
*Modified Rapid Emergency Medicine Score*(mREMS)	2.00 (0.50–4.75)	1.00 (0.00–3.00)	0.103

Data expressed as median and interquartile range. ^M^, *Mann-Whitney test.*

**Figure 1:**
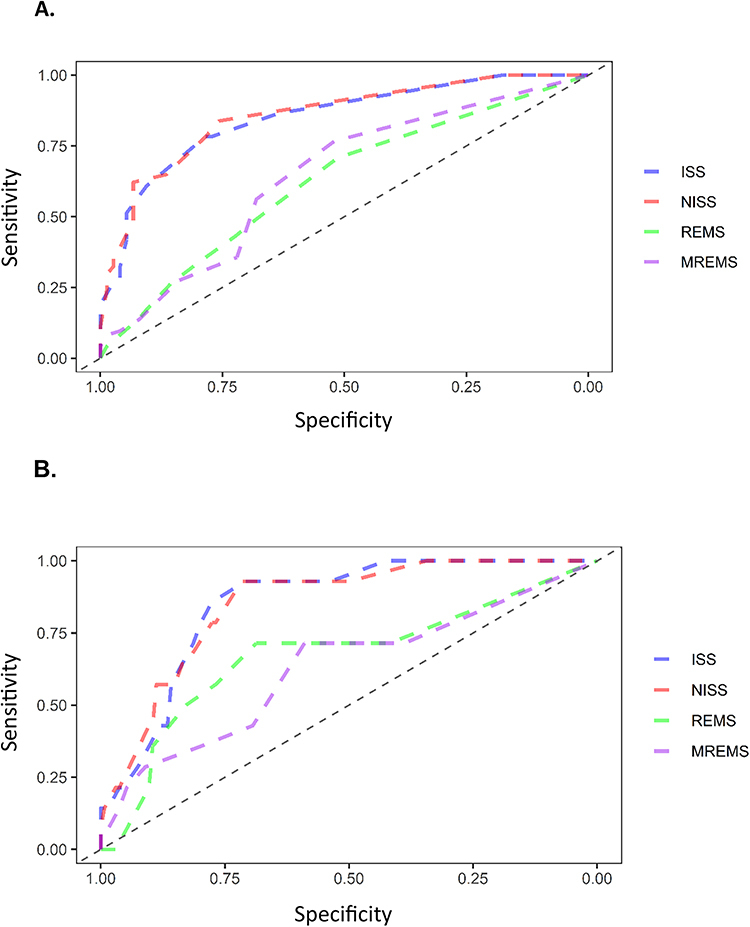
(A). AUC/ROC of the predictive capacity of trauma indices in predicting hospital admission; (B). AUC/ROC of the predictive capacity of trauma indices in predicting ICU admission – Sao Paulo, 2020–2022.

The analysis of the predictive capacity for hospital admission showed that the ISS and NISS performed well in identifying the outcome (AUC/ROC: 0.84; 95%CI = 0.78–0.90 and AUC/ROC: 0.85; 95%CI = 0.78–0.91, respectively). However, the performances of REMS and mREMS were considered unsatisfactory (AUC/ROC: 0.62; 95%CI = 0.53–0.71 and AUC/ROC: 0.64; 95%CI = 0.55–0.73, respectively). Additionally, the 95% CI of the indices indicated that the performance of the ISS and NISS were similar, since they presented overlapping values, but different from the values observed for the REMS and mREMS, which also presented similar performances. The area under the curve is illustrated in Figure 1(A) and the other metrics related to the performance of the indices in relation to the prediction of hospital admission are available in Table 3.

AUC/ROC *Area under curve/Receiver Operating Characteristic Curve*; ISS – *Injury Severity Score*; NISS – *New Injury Severity Score*; REMS – *Rapid Emergency Medicine Score*; mREMS – *modified Rapid Emergency Medicine Score*.

**Table 3:** ROC metrics of severity indices in relation to hospital and ICU admission outcomes – Sao Paulo, 2020–2022.

ROC metrics for hospital admission	Severity indices			
	ISS	NISS	REMS	mREMS
AUC/ROC (95% CI)	0.84 (0.78–0.90)	0.85 (0.79–0.91)	0.62 (0.53–0.71)	0.64 (0.55–0.73)
Cutoff point	3.5	2.5	1	0.5
Sensitivity	0.78	0.83	0.70	0.76
Specificity	0.78	0.75	0.51	0.52
Positive predictive value	0.78	0.77	0.57	0.60
Negative predictive value	0.78	0.82	0.65	0.69
Accuracy	0.78	0.79	0.60	0.64
**ROC metrics for ICU admission**	**ISS**	**NISS**	**REMS**	**mREMS**
AUC/ROC (95% CI)	0.85 (0.77–0.93)	0.85 (0.76–0.93)	0.67 (0.50–0.84)	0.62 (0.45–0.79)
Cutoff point	4.5	5.5	3.5	1.5
Sensitivity	0.92	0.92	0.71	0.71
Specificity	0.70	0.71	0.68	0.58
Positive predictive value	0.25	0.25	0.19	0.15
Negative predictive value	0.98	0.98	0.95	0.95
Accuracy	0.72	0.73	0.68	0.60

AUC/ROC Area under curve/Receiver Operating Characteristic Curve; 95% IC, 95% confidence interval; ISS – Injury Severity Score; NISS – New Injury Severity Score; REMS – Rapid Emergency Medicine Score; mREMS – modified Rapid Emergency Medicine Score. Sample (n = 151); Hospital admission (n = 75), Intensive care admission (n = 14).

Similar to what was observed in relation to hospital admission, the ISS and NISS performed well in discriminating the outcome of ICU admission (AUC/ROC: 0.85, for both; 95%CI = 0.77 – 0.93 and 0.76 – 0.93, respectively). However, the performance of REMS and mREMS was insufficient (AUC: 0.67; 95%CI = 0.50 – 0.84 and AUC/ROC: 0.62; 95%CI = 0.45 – 0.79, respectively), and can be classified as random, according to the values estimated in the 95%CI of AUC/ROC. The AUC representation is shown in Figure 1(B), and the complementary data of the AUC/ROC metrics are shown in Table 3.

## DISCUSSION

Incorporating indices with good performance to predict hospital trajectory and patient prognosis into clinical practice and hospital management can be a valuable strategy to optimize the allocation of human and material resources. Furthermore, its use can contribute to a more structured and personalized approach in the care of trauma patients, aiding in decision-making and improving the quality of care provided. From this perspective, it was found that the ISS and NISS presented good predictive capacity for hospital and ICU admission after admission to the emergency service, while the REMS and mREMS had unsatisfactory performance (AUC/ROC ≤ 0.7) or even random performance, when considering the observed 95% CI (values ≤ 0.6).

It was found that approximately half of the individuals admitted to the emergency service at the study site were referred to hospital inpatient units and, as expected, the median values of the severity indices analyzed were higher (p ≤ 0.05) among those admitted, compared to patients who were discharged from hospital. However, the median ISS and NISS scores among hospitalized patients did not reach values ≥16, indicative of significant trauma^(25,26)^ just as the REMS and mREMS scores were <6, indicating a low risk of hospital mortality for patients^(6,10,13)^.

In decisions related to the care of trauma patients, age and the type of injury diagnosed are relevant factors that may indicate the need for hospital admission, regardless of the severity of the trauma and the physiological changes observed upon admission. The literature has highlighted the relationship between age, traumatic brain injury and worse prognoses^(27)^; consequently, in clinical practice, different approaches are adopted for elderly patients with this type of injury.

Regarding the predictive capacity of severity indices in relation to the outcome of hospital admission of trauma patients, it was observed that the ISS and NISS presented similar performance, as demonstrated by the overlapping 95% CI values. The metrics for the established cutoff points 3.5 for the ISS and 2.5 for the NISS were also close, indicating no difference in performance between the two indices. Based on the formula for calculating these indices (sum of the squares of three AIS scores) and the cutoff points established for hospital admission, it can be deduced that the exclusive presence of mild injuries (AIS 1) discriminated patients who were discharged after being treated in the emergency room, considering that this scale classifies mild injuries with a score of one, moderate injuries with a score of two, serious injuries with a score of three, severe injuries with a score of four, critical injuries with a score of five and maximum severity injuries with a score of six^(20)^.

REMS and mREMS performed worse in discriminating cases that required hospital admission, with unsatisfactory performance in all metrics analyzed, except for sensitivity. According to the 95% CI of AUC/ROC, equivalent performance was observed between REMS and mREMS, but distinct from ISS and NISS.

In this study, the ICU admission rate was lower than that of other Brazilian studies, which reported 11.50%, 24.80% and 81.90% of ICU admission among trauma patients^(15,16,28)^. The median values of the indices were higher among those admitted to the ICU, with a statistically significant difference only for the ISS, NISS, and REMS (p < 0.001 for ISS and NISS; p = 0.027 for REMS). In a study carried out in a supplementary health hospital^(15)^, a statistically significant difference was observed in the values of the three indices (ISS, NISS and mREMS; p < 0.001).

The predictive capacity of severity indices in relation to ICU admission was also evaluated, highlighting the performance of ISS and NISS, both with AUC/ROC of 0.85. Considering cutoff points of 4.5 (ISS) and 5.5 (NISS), similar performance was observed between the two indices, with high sensitivity (0.92), indicating that 92% of patients admitted to the ICU had values above the respective cutoff points. However, the PPV was 0.25 for both indices (ISS and NISS), reflecting the high number of false positives that is, patients with values above the cutoff point who were not admitted to the ICU (n = 39 for ISS and n = 38 for NISS). Only one patient with values below the cutoff points was admitted to the ICU.

On the other hand, REMS and mREMS showed insufficient performance (AUC/ROC of 0.67 and 0.62, respectively), being outperformed by ISS and NISS in all analyses. Corroborating these findings, a previous study indicated that ISS and NISS performed better in predicting ICU admission (AUC/ROC: 0.91; 95% CI = 0.88–0.94) when compared to other indices, such as Revised Trauma Score (RTS), New Trauma Score (NTS), mREMS, Trauma and Injury Severity Score (TRISS), and New Trauma and Injury Severity Score (NTRISS). As in the current study, mREMS showed insufficient performance (AUC/ROC: 0.64; 95% CI = 0.58–0.70) and low positive predictive values, 0.44 (ISS), 0.52 (NISS), and 0.21 (mREMS)^(15)^.

Overall, the results of this study suggest that the REMS and mREMS have a limited role in decisions about patient outcomes after emergency department admission (discharge, hospital admission, and ICU admission), while the ISS and NISS demonstrate potential clinical application in this context. The low PPV of the two indices for ICU admissions highlights the importance of structural conditions that influence this decision, such as the capacity of units to monitor patients and the availability of ICU beds.

In the literature, trauma severity indices based on physiological parameters have been surpassed by anatomical indices in predicting different outcomes^(29)^. Indices such as the ISS and NISS are stable and have a single score, as they exclusively consider injuries diagnosed in trauma in their calculation. On the other hand, indices such as REMS and mREMS that use physiological parameters depend on clinical interventions and post-trauma evolution and vary according to patient care and characteristics of the population studied. Procedures initiated in pre-hospital care can stabilize circulatory and ventilatory conditions, attenuating the severity expressed by indices based on initial vital signs measured in the emergency department.

Despite these aspects, physiological parameters are essential, especially in initial care, when the injuries have not yet been fully diagnosed. In this case, the immediate application of indices such as REMS can help in the early identification of high mortality risk.

Among the limitations of this study, we highlight the sample size and the fact that the population belongs to a private trauma center, with advanced infrastructure and a differentiated patient profile, which limits the generalization of the findings to the public health system. The sample predominantly included low- acuity patients, many of whom were discharged directly from the emergency room.

Furthermore, this is a retrospective study based on initial records from the emergency department, with no possibility of guaranteeing temporal standardization of the collection of physiological parameters, which can impact the accuracy of physiological indices. Moreover, the anatomical indices (ISS and NISS) require a complete diagnosis of the lesions, which often occurs after interventions, limiting their immediate application in initial care. However, this study was conducted in a hospital recognized as a trauma center, with a multidisciplinary team and adequate infrastructure, meeting the technical criteria required for this classification. Although it is a single institution, the hospital serves patients with different conditions of access to supplementary health care, covering different individuals and health insurance plans, which increases the diversity of the population studied.

Studies in this context are fundamental to describe a reality little explored in national and international scientific literature, allowing us to understand the dynamics of trauma care in supplementary healthcare. This contributes to evidence-based decisions, qualified care, and more efficient integration with the public network, especially after the implementation of the Trauma Care Program in Supplementary Health. This integration is strategic to reduce the overload on public hospitals, previously responsible for treating all trauma cases.

## CONCLUSION

The anatomical indices performed well in discriminating individuals who required hospitalization and ICU admission. In contrast, physiological indices showed insufficient performance for these outcomes. It was observed that the ISS and NISS had similar performance to each other, as well as the REMS and mREMS.

The results indicated that the ISS and NISS can be applied clinically in identifying patients requiring hospital and ICU admission, while the use of REMS and mREMS is not recommended for this purpose, based on the findings of this study.

As with other health professionals, for nursing, information on the performance of trauma severity indices provides valuable support for the use of these instruments in clinical practice and hospital management, ensuring patient safety and better outcomes in the care of injured patients.

## Data Availability

The full dataset supporting the findings of this study is available from the corresponding author (Lillian Caroline Fernandes) upon reasonable request.
