# Supplementary material for: Predictive capacity of trauma indices for hospitalization and intensive care unit admission
Source: Rev Esc Enferm USP. 2025 Sep 5;59:e20250092. doi: 10.1590/1980-220X-REEUSP-2025-0092en (PMC12416370; doi:10.1590/1980-220X-REEUSP-2025-0092en)
Supplement: Translation

# Translation: Capacidade preditiva dos índices de trauma para internação hospitalar e admissão em unidade de terapia intensive

**DOI:** 10.1590/1980-220X-REEUSP-2025-0092pt

**Authors:** Lillian Caroline Fernandes, Daniel Bueno Damasceno, Fernanda Naves de Oliveira Lima, Lilia de Souza Nogueira, Ramon Antonio Oliveira, Filipe Utuari de Andrade Coelho, Regina Marcia Cardoso de Sousa

**Affiliations:** 1 Universidade de São Paulo, Escola de Enfermagem, Departamento de Enfermagem Médico-Cirúrgica, São Paulo, SP, Brasil.; 2 Hospital Israelita Albert Einstein, Faculdade de Ciências da Saúde Albert Einstein, São Paulo, SP, Brasil.

**Keywords:** Causas Externas, Índices de Gravidade do Trauma, Unidade de Terapia Intensiva, Enfermagem

## Abstract

**Objetivo:**

Comparar o desempenho dos índices de gravidade do trauma (ISS, NISS, REMS, mREMS) na predição dos desfechos de internação hospitalar e de admissão em Unidade de Terapia Intensiva (UTI).

**Método:**

Estudo de coorte retrospectiva realizada com pacientes atendidos no Pronto-Socorro de hospital privado no período de janeiro 2020 a janeiro de 2022. Foram analisados prontuários de adultos com trauma contuso, penetrante ou misto admitidos até 24 horas após o trauma. Os índices de gravidade foram utilizados para prever a internação hospitalar e a admissão em UTI.

**Resultados:**

A amostra foi composta por 151 pacientes. O ISS e o NISS apresentaram boa capacidade de discriminação para internação hospitalar e UTI (AUC/ROC de 0,84 a 0,85). Já os índices REMS e mREMS mostraram desempenho insuficiente (AUC/ROC de 0,62 a 0,67).

**Conclusão:**

Os índices ISS e NISS foram eficazes na predição de internação hospitalar e UTI, podendo ser utilizados na prática clínica; no entanto, o uso do REMS e mREMS não pode ser recomendado devido ao desempenho insuficiente para esses desfechos.

## INTRODUÇÃO

O trauma é causado pela ação de agentes externos que resultam em lesões ao indivíduo^(1)^ e tem grande relevância mundial devido à sua alta incidência, mortalidade, custos hospitalares e seu potencial de gerar incapacidades temporárias ou permanentes. Dados do Departamento de Informática do Sistema Único de Saúde (DATASUS) demonstram que as causas externas foram responsáveis por 1.557.896 internações em 2024, com taxa de mortalidade de 2,10% e custo total de R$ 2.280.591.137,83 ao Sistema Único de Saúde (SUS). Neste contexto, destaca-se a população de 20–59 anos, que representa cerca de 60,63% dos casos^(2)^.

Com base nesses dados pode-se inferir a importância de atendimento adequado a esse grupo de indivíduos. No trauma, a qualidade do atendimento aos pacientes está diretamente relacionada à eficiência dos profissionais que atuam em seu cuidado desde o atendimento pré-hospitalar até a alta hospitalar, incluindo a reabilitação e reintegração desse indivíduo à sociedade^(3,4)^. Para garantir essa assistência especializada, tanto as instituições públicas quanto as privadas devem dispor de infraestrutura adequada e profissionais capacitados. Nesse propósito, a Portaria nº 1.336 foi estabelecida para organizar os centros de trauma no Brasil, reforçando a necessidade de um sistema estruturado e eficiente^(5)^.

Na implantação desse sistema para que intervenções e triagens imediatas sejam eficazes e para garantir um planejamento adequado da assistência, é essencial que todos os envolvidos no atendimento conheçam a gravidade do quadro clínico de cada paciente^(6)^. Para esse fim, os centros de trauma frequentemente utilizam índices de gravidade do trauma para auxiliar nos processos assistenciais e gerenciais. Essas métricas fornecem uma medida objetiva da gravidade das lesões e estimam desfechos, permitindo um planejamento de ações mais precisas e assertivas^(7,8)^.

Os índices de gravidade do trauma são sistemas que utilizam as alterações anatômicas (índices anatômicos), fisiológicas (índices fisiológicos) ou ambas para sua pontuação. Entre os principais índices fisiológicos estão o *Rapid Emergency Medicine Score* (REMS)^(9)^ e o *Modified Rapid Emergency Medicine Score*(mREMS)^(10)^ e, entre os anatômicos, o *Injury Severity Score* (ISS)^(11)^ e o *New Injury Severity Score* (NISS)^(12)^ têm tido realce.

A internação hospitalar em Unidade de Terapia Intensiva (UTI) e o óbito têm se destacado como desfechos de interesse para pacientes de trauma^(13,14)^. No entanto, não foram encontrados estudos que avaliaram a capacidade preditiva de índices de gravidade do trauma para internação hospitalar, poucos foram os estudos que avaliaram os índices para internação em UTI^(15,16)^ e foram frequentes as pesquisas que analisaram a capacidade prognóstica para óbito^(3,6,16,17)^. A ocorrência de internação hospitalar e admissão em UTI tem despertado interesse pois estão associadas a procedimentos complexos, custos hospitalares e complicações tardias^(13,14)^ após trauma.

A aplicação dos índices de gravidade, associada ao registro detalhado dos eventos traumáticos, tem diversas utilizações clínicas e científicas, contribuindo para melhoria da assistência dos pacientes. Embora existam diversos índices disponíveis, é fundamental identificar aqueles que apresentam maior precisão e confiabilidade em diferentes etapas do atendimento. Além disso, há uma lacuna de estudos voltados à avaliação da capacidade preditiva dos índices de gravidade em relação às internações na UTI e no hospital. Diante desse cenário, este estudo tem como objetivo comparar o desempenho dos índices de trauma (ISS, NISS, REMS e mREMS) na predição desses desfechos.

## MÉTODO

### Desenho do Estudo

Estudo de coorte retrospectiva, redigido de acordo com as recomendações do *STrengthening the Reporting of OBservational studies in Epidemiology* (STROBE)^(18)^, realizado por meio da análise de prontuários eletrônicos de pacientes de trauma admitidos no Pronto-Socorro de um hospital privado no município de São Paulo. Foram coletadas informações sociodemográficas, bem como dados relacionados ao trauma, ao atendimento pré- hospitalar e ao atendimento hospitalar.

### Local do Estudo

O estudo foi realizado em uma instituição hospitalar privada, localizada na região sul da cidade de São Paulo, caracterizada como de porte extra, com mais de 700 leitos ativos. O Pronto-Socorro Adulto desse hospital dispõe de 34 macas e 31 poltronas para o atendimento e cuidado de indivíduos em situação de urgência ou emergência que, a depender das condições clínicas, são encaminhados para unidades específicas de tratamento. A instituição é considerada centro de trauma tipo II, referência para atendimento a pacientes de trauma que têm acesso ao sistema de saúde suplementar ou condições socioeconômicas para arcar com os custos da assistência necessária.

O hospital dispõe de uma equipe de atendimento ao trauma composta por enfermeiro, cirurgião geral e ortopedista, que é acionada pela equipe do Pronto-Socorro com base em critérios fisiológicos, anatômicos e no mecanismo da lesão.

O hospital conta com unidades de UTI adulto distribuídas em dois andares, totalizando 54 leitos ativos. Cada leito é equipado com monitor multiparamétrico contínuo e estrutura para suporte ventilatório, administração de drogas vasoativas, sedação e realização de procedimentos de alta complexidade. Essas unidades possuem perfil assistencial geral, e os pacientes traumatizados são admitidos em qualquer um dos leitos disponíveis.

### Amostra e Critérios de Seleção

A amostra deste estudo foi constituída por pacientes de trauma admitidos no Pronto-Socorro Adulto no período de 1º de janeiro de 2020 a 1º de janeiro de 2022, segundo informações fornecidas pelo Serviço de Arquivo Médico e Estatístico da instituição hospitalar local deste estudo.

Os participantes do estudo atenderam aos seguintes critérios de inclusão: indivíduos com trauma contuso, penetrante ou misto; idade ≥18 anos; admitidos na instituição até 24 horas após o evento traumático. Foram excluídos indivíduos admitidos em parada cardiorrespiratória, pacientes com queimaduras e transferidos de outras instituições, visto que tratamentos anteriores à admissão no local de estudo podem alterar as variáveis fisiológicas utilizadas para o cálculo dos índices de gravidade.

### Coleta de dados

A coleta de dados foi realizada após a aprovação do projeto de pesquisa pelo Comitê de Ética em Pesquisa e pela Coordenação do Pronto-Socorro da instituição. Após as autorizações institucionais, foi solicitado ao Serviço de Arquivo Médico e Estatístico o envio da listagem em que constavam os endereços eletrônicos de todos os atendidos no Pronto-Socorro em consequência de acidentes ou violências, no período de estudo.

Em listagem fornecida por esse serviço, foram identificados, em planilha em formato Microsoft Excel, com acesso restrito via rede interna e e-mail corporativo, os números dos prontuários e os endereços eletrônicos dos pacientes admitidos por causas externas no setor de emergência, durante o período estabelecido na pesquisa.

Por meio dessa listagem, foram contatados os sujeitos elegíveis por correio eletrônico institucional, com o objetivo de convidá-los a participar da pesquisa. O e-mail enviado continha, em anexo, o instrumento de coleta de dados e o link para acesso ao Termo de Consentimento Livre e Esclarecido (TCLE). Na ausência de repostas a essa abordagem, foram feitas novas tentativas de contato e enviados até cinco e-mails com esse conteúdo.

Os dados foram coletados no período de julho de 2023 a julho de 2024, por meio de consulta ao prontuário eletrônico, após a assinatura do TCLE. As variáveis dependentes foram a internação hospitalar e a admissão em UTI. As variáveis independentes foram os índices de gravidade: REMS, mREMS, ISS e NISS.

O REMS é um indicador fisiológico de gravidade que é composto pela Escala de Coma de Glasgow (ECGl), frequência cardíaca (FC), pressão arterial média (PAM), frequência respiratória (FR), saturação de oxigênio (SatO_2_) e idade. Conforme os valores observados na admissão no serviço de emergência, essas variáveis recebem pontuação de zero a quatro pontos, exceto a idade, que varia de zero a seis^(9)^. O REMS foi desenvolvido para ser aplicado em pacientes não cirúrgicos admitidos na emergência; no entanto, tem sido demonstrada sua aplicabilidade em pacientes de trauma^(19)^.

Com objetivo de melhorar sua capacidade preditiva de desfechos na população de trauma, em 2017 foi desenvolvido o mREMS^(10)^. Nesse instrumento, foi alterada a ponderação das variáveis idade, ECGl e a PAM foi substituída pela pressão arterial sistólica (PAS). Para o mREMS, a pontuação da ECGl varia de 0 a 6 e os demais parâmetros pontuam de 0 a 4^(10,12)^. A somatória da pontuação obtida nas variáveis do REMS e do mREMS resulta nos escores dos índices que variam de zero a 26, sendo que as maiores pontuações indicam maior gravidade.

O cálculo do ISS^(11)^ e do NISS^(12)^ segue as diretrizes do *Abbreviated Injury Scale* (AIS)^(20)^, manual que apresenta uma lista com descrição de inúmeras lesões, utilizada para identificar cada lesão do paciente e sua gravidade. Essa lista fornece, para cada descrição de lesão, um identificador composto por sete números, sendo o último dígito o valor referente à gravidade da lesão. O ISS e o NISS utilizam em seu cálculo a gravidade das lesões estabelecida pela AIS e, por métodos específicos de cálculo, estimam a gravidade global de um paciente^(12)^. A pontuação do ISS e do NISS varia de 1 a 75, sendo que um AIS de 6 atribui automaticamente um ISS de 75, sugerindo um quadro fatal.

Para caracterização da amostra estudada foram coletadas variáveis sociodemográficas (sexo e idade) e informações relacionadas ao evento traumático (mecanismo do trauma; causa externa; atendimento pré-hospitalar; tipo de suporte; e transporte pré-hospitalar). Da admissão hospitalar, foram analisados parâmetros clínicos e, do período de internação hospitalar, foram consideradas as abordagens cirúrgicas, o destino após o atendimento no Pronto-Socorro e o tempo de permanência hospitalar.

Os dados foram coletados por meio do instrumento de coleta de dados, e foi elaborada uma ficha de registro das lesões para o cálculo dos índices de gravidade anatômicos.

### Análise e Tratamento dos Dados

Todas as informações coletadas foram armazenadas em banco de dados computadorizado, protegido com senha, de forma a garantir a segurança da informação. Além disso, todos os dados passíveis de identificação foram codificados e anonimizados. O *Software Jamovi*® versão 2.3.26.0 foi utilizado para as análises estatísticas.

As causas externas identificadas nos prontuários dos participantes deste estudo foram categorizadas de acordo com o Capítulo XX – Causas externas de morbidade e de mortalidade (V01-Y98) da Classificação Internacional de Doenças, 10ª Revisão (CID 10)^(21)^. Nos casos em que o trauma não pôde ser especificado nesse capítulo, utilizou-se código CID T14.9, correspondente a “trauma não especificado”, pertencente ao Capítulo XIX dessa classificação.

O REMS e mREMS foram calculados a partir dos dados fisiológicos registrados no atendimento inicial dos pacientes admitidos no Pronto-Socorro. Os escores desses índices foram estabelecidos pela somatória da pontuação atribuída às variáveis fisiológicas que compõem os instrumentos^(9,10)^.

Para o cálculo do ISS, foi realizada a soma dos quadrados dos escores AIS mais altos das três regiões corporais mais afetadas no trauma^(11)^. Para determinar o ISS, foram identificadas as lesões de maior gravidade em cada uma das regiões afetadas e selecionaram-se os três maiores escores AIS em regiões distintas. O cálculo foi realizado somando o quadrado desses três valores: ISS = (AIS1)^2^ + (AIS2)^2^ + (AIS3)^2^. Para estabelecer o valor do NISS, foram somados os quadrados dos três escores AIS mais altos, independentemente da região corporal afetada^(12)^.

Para estabelecer os valores do ISS e do NISS, todas as lesões dos pacientes foram consideradas. Ressalta-se que os índices anatômicos foram calculados em duplicidade e de forma independente. Em caso de discordância entre os avaliadores, um terceiro membro, com ampla experiência em codificação de lesões, foi consultado. Ao final dessa etapa, todas as codificações e cálculos foram revisados e validados por esse terceiro membro.

Foi realizado o teste de *Shapiro-Wilk* para verificar a distribuição dos dados e, visto que não apresentaram distribuição normal, o teste de *Mann-Whitney* foi aplicado nas comparações de grupos de pacientes internados ou não em unidades do hospital e em UTI*.* Para avaliação da capacidade preditiva dos índices de trauma, foram construídas curvas *Receiver Operating Characteristic* (curvas ROC) para os desfechos de interesse do estudo (internação hospitalar e admissão em UTI).

O Intervalo de Confiança de 95% da área sob a curva ROC (AUC/ROC) foi utilizado para inferir diferenças estatisticamente significativas entre valores das AUC/ROC^(22)^. De forma complementar, foi realizado o índice de *Youden* para seleção do ponto de corte e obtidas as medidas de sensibilidade, especificidade, valor preditivo positivo (VPP), valor preditivo negativo (VPN) e acurácia.

A categorização dos valores das métricas da AUC/ROC seguiu o formato proposto na literatura científica: ≤0,6 indicação de comportamento aleatório; >0,6 e ≤0,7 capacidade de discriminação insuficiente; >0,7 e ≤0,8 foi considerado aceitável; >0,8 e ≤0,9 boa capacidade de discriminação e; >0,9 excelente^(23)^. O nível de significância adotado em todas as análises foi de 5%.

### Aspectos Éticos

Para a realização deste estudo, foram respeitadas as Diretrizes e Normas Regulamentadoras de Pesquisas envolvendo seres humanos, emanadas da Resolução N.º 466/12 do Conselho Nacional de Saúde^(24)^. O projeto foi submetido e aprovado pelo Comitê de Ética em Pesquisa (CEP), via plataforma Brasil (parecer n. 6.107.403 – CAAE 68381923.4.0000.0071).

O TCLE foi aplicado por meio eletrônico. Inicialmente, os pesquisadores entraram em contato com os potenciais participantes do estudo via e-mail, para convidá-los a participar. Junto ao texto do convite, foram apresentados os arquivos de coleta de dados em formato PDF e o link para acesso ao TCLE, disponibilizado via REDCap® (Vanderbilt, Nashville, TN).

Após assinatura, foi enviada ao paciente e/ou familiar uma cópia dos documentos para eventuais consultas posteriores. Nessa oportunidade, os pesquisadores orientaram quanto à importância de arquivar a cópia e informaram aos participantes que poderiam retirar seu consentimento em qualquer etapa do desenvolvimento do estudo. Caso isso ocorresse, seria enviado um e-mail comprovando a retirada de suas informações do estudo. O TCLE foi aplicado preferencialmente ao paciente e, apenas em casos de comprometimento da compreensão do termo ou em caso de óbito, foi aplicado ao familiar.

## RESULTADOS

Durante o período do estudo, 264 pacientes foram atendidos no Pronto-Socorro em decorrência de acidentes ou violências. Após a aplicação dos critérios de elegibilidade, 42 pacientes (15,90%) foram excluídos, sendo 31 por idade inferior a 18 anos (11,74%), nove por terem sido transferidos de outras instituições (3,40%) e dois por causa externa específica (queimadura) (0,75%). Dessa forma, 222 pacientes com trauma atenderam aos critérios de elegibilidade (84,09%).

Para esses pacientes, foi enviado, via e-mail, o TCLE, solicitando consentimento para uso de seus registros hospitalares na pesquisa. Contudo, 71 pacientes (31,98%) não responderam após cinco tentativas de contato ou recusaram participar. Assim, a amostra foi composta por 151 participantes. Dentre eles, 75 pacientes (49,66%) tiveram indicação de internação hospitalar e 14 (9,27%) foram admitidos em UTI.

A média de idade dos pacientes foi de 52,40 anos, a mediana foi de 47 anos, com valores mínimos e máximos de 19 e 100 anos, respectivamente. A maior frequência foi do sexo masculino (55%), e o trauma contuso foi o principal mecanismo das lesões (95,40%). Dentre as causas externas, quedas e acidentes envolvendo ciclistas traumatizados apresentaram a maior incidência (43,71% e 16,56%, respectivamente).

Referente ao atendimento pré-hospitalar, 57% dos participantes não receberam esse tipo de atendimento; os demais receberam, com maior frequência, atendimento básico (37,10%) por equipes terrestres (41,70%), conforme demonstrado na Tabela 1.

**Tabela 1:** Dados relacionados ao trauma, ao atendimento hospitalar e índices de gravidade do trauma dos participantes da pesquisa (n = 151) – São Paulo, 2020–2022.

Variáveis relacionadas ao trauma	
Mecanismo do trauma – n (%)	
Contuso	144 (95,40)
Penetrante	7 (4,60)
Causa externa / código CID10 – n (%)	
Quedas / W00-W19	66 (43,71)
Ciclista traumatizado / V10-V19	25 (16,56)
Motociclista traumatizado / V20-V29	17 (11,26)
Ocupante de um automóvel traumatizado / V40-V49	15 (9,93)
Pedestre traumatizado / V01-V09	6 (3,97)
Exposição a forças mecânicas inanimadas / W20-W49	6 (3,97)
Outros acidentes de transporte terrestre /V80-V89	4 (2,65)
Outros traumas* / V30-V39; V90-V94; X85-Y09; X58-X59; X60 X84	5 (3,31)
Trauma não especificado / T14.9	7 (4,64)
Tipo de suporte pré-hospitalar – n (%)	
Básico	56 (37,10)
Intermediário	5 (3,30)
Avançado	4 (2,60)
Sem suporte	86 (57,00)
Tipo de transporte pré-hospitalar – n (%)	
Aéreo	2 (1,30)
Terrestre	63 (41,70)
Não se aplica	86 (57,00)
Sinais vitais na admissão do Pronto Socorro – mediana (Q1–Q3)	
Frequência cardíaca, batimentos por minuto	77,00 (69,00–89,00)
Pressão Arterial Sistólica, milímetros de mercúrio	123,00 (105–142)
Pressão Arterial Diastólica, milímetros de mercúrio	85,00 (74,50–99,50)
Pressão Arterial Média, milímetros de mercúrio	97,70 (88,20–109,00)
Escala de Coma de Glasgow	15,00 (15,00–15,00)
Saturação periférica de oxigênio, %	98,00 (96,00–99,00)
Temperatura axilar, graus celsius	36,1 (36,00–36,50)
**Variáveis relacionadas ao atendimento hospitalar**	
Destino após atendimento na sala de emergência – n (%)	
Alta	76 (50,33)
Centro Cirúrgico	25 (16,56)
Observação no Pronto-Socorro	1 (0,66)
Unidade de Internação	31 (20,53)
Unidade Semi-Intensiva	4 (2,65)
Unidade de Terapia Intensiva	14 (9,27)
Abordagem cirúrgica – n (%)	
Sim	51 (33,80)
Não	100 (66,20)
Transferência hospitalar – n (%)	
Sim	13 (8,60)
Não	138 (91,40)
Desfecho – n (%)	
Sobrevivente	150 (99,30)
Óbito	1 (0,70)
Tempo de permanência hospitalar – em dias	
Mediana (Q1–Q3)	1,00 (0,00–3,00)
Índice de gravidade do trauma, mediana (Q1–Q3)	
*Injury Severity Score*(ISS)	3,50 (1,00–8,00)
*New Injury Severity Score*(NISS)	3,50 (1,00–9,00)
*Rapid Emergency Medicine Score*(REMS)	2,00 (0,00–5,00)
*Modified Rapid Emergency Medicine Score*(mREMS)	1,00 (0,00–3,00)

Dados expressos em número e porcentagem (%) ou em mediana e quartil 1 (25%), Q1 e quartil 3 (75%), Q3. (*) Outros traumas: ocupante de um triciclo / V30-V39 (n = 1); acidentes de transporte por água / V90-V94 (n = 1); exposição acidental a outros fatores ou não especificado / X58-X59 (n = 1); lesões autoprovocadas / X60-X84 (n = 1); agressão / X85-Y09 (n = 1).

Verificou-se que 2,71% dos participantes apresentaram algum tipo de alteração nos parâmetros clínicos na admissão do Pronto-Socorro. A abordagem cirúrgica foi indicada para 51 pacientes (33,80%), e 13 participantes do estudo (8,60%) foram transferidos para outro hospital. O tempo mediano de permanência hospitalar foi de 1,00 dia (Q1: 0,00 – Q3: 3,00) e somente um óbito hospitalar foi constatado. Os valores medianos dos índices de gravidade estão descritos na Tabela 1.

Conforme apresentado na Tabela 2, os pacientes que necessitaram de internação hospitalar apresentaram valores mais elevados nos índices de gravidade do trauma em comparação àqueles que não foram internados. Foi verificada diferença estatisticamente significativa entre os grupos com e sem internação hospitalar em relação aos índices analisados. Na comparação entre os pacientes internados ou não em UTI, foram observados valores mais elevados entre aqueles internados nessa unidade. Houve diferença estatisticamente significativa nos valores de ISS (p < 0,001), NISS (p < 0.001), REMS (p = 0,027); no entanto, não foi observada diferença estatisticamente significativa nos valores do mREMS (p = 0,103).

**Tabela 2:** Comparação dos índices de gravidade dos pacientes de trauma com e sem internação hospitalar e admissão em UTI – São Paulo, 2020–2022.

Índice de gravidade do trauma, mediana (Q1–Q3)	Internação hospitalar		p^M^
	Sim (n = 75)	Não (n = 76)	
*Injury Severity Score*(ISS)	6,00 (4,00–13,00)	1,00 (1,00–2,00)	<0,001
*New Injury Severity Score*(NISS)	6,00 (4,00–15,00)	1,00 (1,00–2,00)	<0,001
*Rapid Emergency Medicine Score*(REMS)	2,00 (0,00–6,00)	0,00 (0,00–4,00)	0,007
*Modified Rapid Emergency Medicine Score*(mREMS)	2,00 (1,00–4,00)	0,00 (0,00–3,00)	0,002
**Índice de gravidade do trauma, mediana (Q1–Q3)**	**Admissão em UTI**		**p^M^**
	**Sim (n = 14)**	**Não (n = 137)**	
*Injury Severity Score*(ISS)	10,00 (8,25–13,80)	2,00 (1,00–5,00)	<0,001
*New Injury Severity Score*(NISS)	14,00 (9,00–17,00)	2,00 (1,00–6,00)	<0,001
*Rapid Emergency Medicine Score*(REMS)	5,50 (1,00–7,00)	2,00 (1,00–4,00)	0,027
*Modified Rapid Emergency Medicine Score*(mREMS)	2,00 (0,50–4,75)	1,00 (0,00–3,00)	0,103

Dados expressos em mediana e intervalo interquartil. M, teste de *Mann-Whitney.*

A análise da capacidade preditiva de internação hospitalar mostrou que o ISS e o NISS apresentaram bom desempenho na identificação do desfecho (AUC/ROC: 0,84; IC95% = 0,78 – 0,90 e AUC/ROC: 0,85; IC95% = 0,78 – 0,91, respectivamente). No entanto, os desempenhos do REMS e do mREMS foram considerados insatisfatórios (AUC/ROC: 0,62; IC95% = 0,53 – 0,71 e AUC/ROC: 0,64; IC95% = 0,55 – 0,73, respectivamente). Adicionalmente, os IC95% dos índices indicaram que o desempenho do ISS e do NISS foram semelhantes, uma vez que apresentaram valores sobrepostos, porém diferentes dos valores observados para o REMS e o mREMS, que também apresentaram desempenhos semelhantes entre si. A área sobre a curva está ilustrada na Figura 1(A) e as demais métricas relacionadas ao desempenho dos índices em relação à predição de internação hospitalar estão disponíveis na Tabela 3.

**Figura 1:** (A). AUC/ROC da capacidade preditiva dos índices de trauma na predição de internação hospitalar; (B). AUC/ROC da capacidade preditiva dos índices de trauma na predição de internação em UTI – São Paulo, 2020–2022.

AUC/ROC – *Area under curve/Receiver Operating Characteristic Curve*; ISS – *Injury Severity Score*; NISS – *New Injury Severity Score*; REMS – *Rapid Emergency Medicine Score*; mREMS – *modified Rapid Emergency Medicine Score*.

**Tabela 3:** Métricas da ROC dos índices de gravidade em relação aos desfechos internação hospitalar e em UTI – São Paulo, 2020–2022.

Métricas da ROC para internação hospitalar	Índices de gravidade			
	ISS	NISS	REMS	mREMS
AUC/ROC (IC 95%)	0,84 (0,78–0,90)	0,85 (0,79–0,91)	0,62 (0,53–0,71)	0,64 (0,55–0,73)
Ponto de corte	3,5	2,5	1	0,5
Sensibilidade	0,78	0,83	0,70	0,76
Especificidade	0,78	0,75	0,51	0,52
Valor preditivo positivo	0,78	0,77	0,57	0,60
Valor preditivo negativo	0,78	0,82	0,65	0,69
Acurácia	0,78	0,79	0,60	0,64
**Métricas da ROC para internação em UTI**	**ISS**	**NISS**	**REMS**	**mREMS**
AUC/ROC (IC 95%)	0,85 (0,77–0,93)	0,85 (0,76–0,93)	0,67 (0,50–0,84)	0,62 (0,45–0,79)
Ponto de corte	4,5	5,5	3,5	1,5
Sensibilidade	0,92	0,92	0,71	0,71
Especificidade	0,70	0,71	0,68	0,58
Valor preditivo positivo	0,25	0,25	0,19	0,15
Valor preditivo negativo	0,98	0,98	0,95	0,95
Acurácia	0,72	0,73	0,68	0,60

AUC/ROC – *Area* under *curve*/*Receiver Operating Characteristic Curve*; IC 95%, intervalo de confiança de 95%; ISS – *Injury Severity Score*; NISS – *New Injury Severity Score*; REMS – *Rapid Emergency Medicine Score*; mREMS – *modified Rapid Emergency Medicine Score.* Amostra (n = 151); Internação hospitalar (n = 75), Internação em terapia intensiva (n = 14).

Similarmente ao observado em relação a internação hospitalar, o ISS e o NISS apresentaram bom desempenho na discriminação do desfecho internação em UTI (AUC/ROC: 0,85, para ambos; IC95%= 0,77 – 0,93 e 0,76 – 0,93, respectivamente). Entretanto, o desempenho do REMS e do mREMS foi insuficiente (AUC: 0,67; IC95% = 0,50 – 0,84 e AUC/ROC: 0,62; IC95% = 0,45 – 0,79, respectivamente), podendo ser classificado como aleatório, segundo os valores estimados no IC95% da AUC/ROC. A representação da AUC está apresentada na Figura 1(B), e os dados complementares das métricas da AUC/ROC estão dispostos na Tabela 3.

## DISCUSSÃO

A incorporação dos índices com bom desempenho para prever a trajetória hospitalar e o prognóstico dos pacientes na prática clínica e na gestão hospitalar pode ser uma estratégia valiosa para otimizar a alocação de recursos humanos e materiais. Além disso, seu uso pode contribuir para uma abordagem mais estruturada e personalizada no atendimento a pacientes com trauma, auxiliando na tomada de decisões e aprimorando a qualidade da assistência prestada. Dentro dessa perspectiva, verificou-se que o ISS e o NISS apresentaram boa capacidade preditiva de internação hospitalar e em UTI após a admissão em serviço de emergência, enquanto o REMS e o mREMS tiveram desempenho insatisfatório (AUC/ROC ≤0,7) ou até aleatório, quando considerado o IC95% observado (valores ≤ 0,6).

Foi verificado que aproximadamente metade dos indivíduos admitidos no serviço de emergência do local do estudo foram encaminhados para unidades de internação do hospital e, conforme esperado, os valores medianos dos índices de gravidade analisados foram superiores (p ≤ 0,05) entre os internados, em comparação com os pacientes que receberam alta hospitalar. Entretanto, a pontuação mediana do ISS e do NISS entre os pacientes internados não atingiu valores ≥16, indicativo de trauma importante^(25,26)^ assim como as pontuações do REMS e do mREMS foram <6, apontando baixo risco de mortalidade hospitalar dos pacientes^(6,10,13)^.

Nas decisões relacionadas ao atendimento de pacientes traumatizados, a idade e o tipo de lesão diagnosticada são fatores relevantes que podem indicar a necessidade de internação hospitalar, independentemente da gravidade do trauma e das alterações fisiológicas observadas na admissão. A literatura tem destacado a relação entre idade, trauma cranioencefálico e piores prognósticos^(27)^; em consequência, observa-se na prática clínica a adoção de condutas diferenciadas para pacientes idosos e com esse tipo de lesão.

Referente à capacidade preditiva dos índices de gravidade em relação ao desfecho de internação hospitalar de pacientes com trauma, observou-se que o ISS e o NISS apresentaram desempenho semelhante, como demonstrado pelos valores de IC95% sobrepostos. As métricas para os pontos de corte estabelecidos 3,5 para o ISS e 2,5 para o NISS também foram próximas, não indicando diferença no desempenho entre os dois índices. Com base na fórmula de cálculo desses índices (soma dos quadrados de três escores AIS) e nos pontos de corte estabelecidos para internação hospitalar, pode-se deduzir que a presença exclusiva de lesões leves (AIS 1) discriminou os pacientes que receberam alta após o atendimento na sala de emergência, considerando que essa escala classifica lesões leves com escore um, moderadas com escore dois, sérias com três, graves com quatro, críticas com cinco e de gravidade máxima com seis^(20)^.

O REMS e mREMS apresentaram pior desempenho na discriminação de casos que necessitaram de internação hospitalar, com desempenho insatisfatório em todas as métricas analisadas, exceto na sensibilidade. Pelos IC95% da AUC/ROC, observou- se desempenho equivalente entre o REMS e o mREMS, porém distinto do ISS e do NISS.

Neste estudo, a taxa de internação na UTI foi inferior à de outros estudos brasileiros, que relataram 11,50%, 24,80% e 81,90% de admissão em UTI entre os pacientes com trauma^(15,16,28)^. Os valores medianos dos índices foram mais elevados entre os internados em UTI, com diferença estatisticamente significativa apenas para o ISS, NISS e REMS (p < 0,001 para ISS e NISS; p = 0,027 para o REMS). Em estudo realizado em um hospital da saúde suplementar^(15)^, foi observada diferença estatisticamente significativa nos valores dos três índices (ISS, NISS e mREMS; p < 0,001).

A capacidade preditiva dos índices de gravidade em relação à admissão em UTI também foi avaliada, destacando-se o desempenho do ISS e do NISS, ambos com AUC/ROC de 0,85. Considerando pontos de corte de 4,5 (ISS) e 5,5 (NISS), observou-se desempenho semelhante entre os dois índices, com alta sensibilidade (0,92), indicando que 92% dos pacientes admitidos na UTI apresentaram valores acima dos respectivos pontos de corte. No entanto, o VPP foi de 0,25 para ambos os índices (ISS e NISS), reflexo do elevado número de falso positivos ou seja, pacientes com valores acima do ponto de corte que não foram admitidos na UTI (n = 39 para ISS e n = 38 para NISS). Apenas um paciente com valores inferiores aos pontos de corte foi admitido em UTI.

Por outro lado, o REMS e o mREMS apresentaram desempenho insuficiente (AUC/ROC de 0,67 e 0,62, respectivamente), sendo superados pelo ISS e NISS em todas as análises. Corroborando esses achados, estudo prévio indicou que ISS e o NISS tiveram melhor desempenho na predição de internação em UTI (AUC/ROC: 0,91; IC95% = 0,88–0,94) quando comparados a outros índices, como Revised Trauma Score (RTS), New Trauma Score (NTS), mREMS, Trauma and Injury Severity Score (TRISS) e New Trauma and Injury Severity Score (NTRISS). Assim como no atual estudo, o mREMS apresentou desempenho insuficiente (AUC/ROC: 0,64; IC95% = 0,58–0,70) e baixos valores preditivos positivos, 0,44 (ISS), 0,52 (NISS) e 0,21(mREMS)^(15)^.

De modo geral, os resultados deste estudo sugerem que o REMS e o mREMS têm papel limitado nas decisões sobre a trajetória dos pacientes após a admissão no serviço de emergência (alta, internação hospitalar e admissão na UTI), enquanto o ISS e o NISS demonstram potencial aplicação clínica nesse contexto. O baixo VPP dos dois índices para internação em UTI alerta sobre a importância de condições estruturais que pesam nessa decisão, tais como capacidade das unidades para monitorização dos pacientes e vagas de leitos na UTI.

Na literatura, índices de gravidade do trauma baseados em parâmetros fisiológicos têm sido superados por índices anatômicos na previsão de diferentes desfechos^(29)^. Índices como o ISS e o NISS são estáveis, apresentam pontuação única, por considerarem em seu cálculo exclusivamente as lesões diagnosticadas no trauma. Por outro lado, índices como o REMS e o mREMS que utilizam parâmetros fisiológicos dependem de intervenções clínicas e da evolução pós trauma e variam conforme o atendimento aos pacientes e características da população estudada. Procedimentos iniciados no atendimento pré-hospitalar podem estabilizar as condições circulatórias e ventilatórias, atenuando a gravidade expressa por índices baseados em sinais vitais iniciais mensurados no departamento de emergência.

Apesar desses aspectos, parâmetros fisiológicos são essenciais, sobretudo no atendimento inicial, quando as lesões ainda não foram plenamente diagnosticadas. Nesse caso, a aplicação imediata de índices como o REMS pode auxiliar na identificação precoce de risco elevado de mortalidade.

Entre as limitações deste estudo, destaca-se o tamanho amostral e o fato de a população pertencer a um centro de trauma privado, com infraestrutura avançada e perfil de pacientes diferenciado, o que limita a generalização dos achados para o sistema público de saúde. A amostra incluiu predominantemente pacientes com baixa gravidade, muito dos quais receberam alta diretamente da sala de emergência.

Além disso, trata-se de um estudo retrospectivo baseado em registros iniciais do departamento de emergência, sem possibilidade de garantir a padronização temporal da coleta de parâmetros fisiológicos, o que pode impactar a acurácia dos índices fisiológicos. Além disso, os índices anatômicos (ISS e NISS) demandam diagnóstico completo das lesões, o que frequentemente ocorre após intervenções, limitando sua aplicação imediata no atendimento inicial. No entanto, este estudo foi conduzido em hospital reconhecido como centro de trauma, com equipe multiprofissional e infraestrutura adequada, atendendo aos critérios técnicos exigidos para essa classificação. Embora se trate de uma única instituição, o hospital atende pacientes com diferentes condições de acesso à saúde suplementar, abrangendo diversos indivíduos e convênios de saúde, o que amplia a diversidade da população estudada.

Estudos nesse contexto são fundamentais para descrever uma realidade pouco explorada na literatura científica nacional e internacional, permitindo compreender a dinâmica do atendimento ao trauma na saúde suplementar. Isso contribui para decisões baseadas em evidências, qualificação do cuidado e integração mais eficiente com a rede pública, sobretudo após a implementação do Programa de Atendimento ao Trauma na Saúde Suplementar. Essa integração é estratégica para reduzir a sobrecarga dos hospitais públicos, anteriormente responsáveis pelo atendimento de todos os casos de trauma.

## CONCLUSÃO

Os índices anatômicos apresentaram bom desempenho para discriminar os indivíduos que necessitaram de internação hospitalar e em UTI. Em contrapartida, os índices fisiológicos apresentaram desempenho insuficiente para esses desfechos. Observou-se que o ISS e o NISS tiveram desempenho semelhante entre si, assim como o REMS e o mREMS.

Os resultados indicaram que o ISS e o NISS podem ser aplicados clinicamente na identificação de pacientes com necessidade de internação hospitalar e em UTI, enquanto o uso do REMS e do mREMS não é recomendado para essa finalidade, com base nos achados deste estudo.

Assim como para outros profissionais da saúde, para a enfermagem, as informações sobre o desempenho dos índices de gravidade do trauma trazem subsídios valiosos para o uso desses instrumentos na prática clínica e gestão hospitalar, garantindo segurança ao paciente e melhores desfechos no atendimento a pacientes com trauma.

## Data Availability

Todo o conjunto de dados que dá suporte aos resultados deste estudo está disponível mediante solicitação ao autor correspondente (Lillian Caroline Fernandes).

# Translation: Capacidad predictiva de los índices de trauma para la hospitalización y el ingreso a la unidad de cuidados intensivos

**DOI:** 10.1590/1980-220X-REEUSP-2025-0092es

**Authors:** Lillian Caroline Fernandes, Daniel Bueno Damasceno, Fernanda Naves de Oliveira Lima, Lilia de Souza Nogueira, Ramon Antonio Oliveira, Filipe Utuari de Andrade Coelho, Regina Marcia Cardoso de Sousa

**Affiliations:** 1 Universidade de São Paulo, Escola de Enfermagem, Departamento de Enfermagem Médico-Cirúrgica, São Paulo, SP, Brasil.; 2 Hospital Israelita Albert Einstein, Faculdade de Ciências da Saúde Albert Einstein, São Paulo, SP, Brasil.

**Keywords:** Causas Externas, Índices de Gravedad del Trauma, Unidades de Cuidados Intensivos, Enfermería

## Abstract

**Objetivo:**

Comparar el desempeño de los índices de gravedad del trauma (ISS, NISS, REMS, mREMS) para predecir los resultados de admisión hospitalaria y a la Unidad de Cuidados Intensivos (UCI).

**Método:**

Estudio de cohorte retrospectivo realizado con pacientes atendidos en el Servicio de Urgencias de un hospital privado desde enero de 2020 a enero de 2022. Se analizaron los registros médicos de adultos con traumatismo cerrado, penetrante o mixto ingresados hasta 24 horas después del trauma. Se utilizaron índices de gravedad para predecir el ingreso hospitalario y en la UCI.

**Resultados:**

La muestra estuvo constituida por 151 pacientes. El ISS y el NISS mostraron una buena capacidad de discriminación para el ingreso hospitalario y en UCI (AUC/ROC de 0,84 a 0,85). Los índices REMS y mREMS mostraron un rendimiento insuficiente (AUC/ROC de 0,62 a 0,67).

**Conclusión:**

Los índices ISS y NISS fueron eficaces para predecir la admisión en hospitales y UCI y pueden utilizarse en la práctica clínica; sin embargo, no se puede recomendar el uso de REMS y mREMS debido al rendimiento insuficiente para estos resultados.

## INTRODUCCIÓN

El trauma es causado por la acción de agentes externos que provocan lesiones al individuo^(1)^ y tiene gran relevancia mundial debido a su alta incidencia, mortalidad, costos hospitalarios y su potencial para generar discapacidades temporales o permanentes. Los datos del Departamento de Informática del Sistema Único de Saúde (DATASUS) muestran que las causas externas fueron responsables de 1.557.896 hospitalizaciones en 2024, con una tasa de mortalidad del 2,10 % y un coste total de 2.280.591.137,83 reales brasileños para el Sistema Único de Saúde (SUS). En este contexto, destaca la población de 20 a 59 años, que representa alrededor del 60,63 % de los casos^(2)^.

A partir de estos datos, se puede inferir la importancia de una atención adecuada a este grupo de personas. En traumatología, la calidad de la atención a los pacientes está directamente relacionada con la eficiencia de los profesionales que los atienden desde la asistencia prehospitalaria hasta el alta hospitalaria, incluyendo la rehabilitación y la reintegración de estas personas en la sociedad^(3,4)^. Para garantizar esta asistencia especializada, tanto las instituciones públicas como las privadas deben disponer de una infraestructura adecuada y de profesionales capacitados. Con este fin, se estableció la Ordenanza n.º 1.336 para organizar los centros de traumatología en Brasil, reforzando la necesidad de un sistema estructurado y eficiente^(5)^.

Para que este sistema funcione y las intervenciones y la clasificación inmediatas sean eficaces, y para garantizar una planificación adecuada de la asistencia, es esencial que todos los involucrados en la atención conozcan la gravedad del cuadro clínico de cada paciente^(6)^. Con este fin, los centros de traumatología suelen utilizar índices de gravedad del trauma para ayudar en los procesos asistenciales y de gestión. Estas métricas proporcionan una medida objetiva de la gravedad de las lesiones y estiman los resultados, lo que permite planificar acciones más precisas y acertadas^(7,8)^.

Los índices de gravedad del trauma son sistemas que utilizan alteraciones anatómicas (índices anatómicos), fisiológicas (índices fisiológicos) o ambas para su puntuación. Entre los principales índices fisiológicos se encuentran el Escore rápido de urgencias (*Rapid Emergency Medicine Score* REMS)^(9)^ y el Escore rápido de urgencias modificado (*Modified Rapid Emergency Medicine Score* mREMS)^(10)^ y, entre los anatómicos, el Índice de gravedad de la lesión (*Injury Severity Score*- ISS)^(11)^ y el Nuevo Índice de gravedad de la lesión (*New Injury Severity Score* NISS)^(12)^ han cobrado relevancia.

La hospitalización en una Unidad de Terapia Intensiva (UTI) y la muerte se han destacado como resultados de interés para los pacientes traumatizados^(13,14)^. Sin embargo, no se han encontrado estudios que evalúen la capacidad predictiva de los índices de gravedad del trauma para la hospitalización, pocos estudios han evaluado los índices para la admisión en la UTI^(15,16)^ y han sido frecuentes las investigaciones que han analizado la capacidad pronóstica para la muerte^(3,6,16,17)^. La hospitalización y el ingreso en la UTI han despertado interés, ya que están asociados a procedimientos complejos, costes hospitalarios y complicaciones tardías^(13,14)^ tras un traumatismo.

La aplicación de índices de gravedad, junto con el registro detallado de los eventos traumáticos, tiene diversos usos clínicos y científicos, lo que contribuye a mejorar la atención de los pacientes. Aunque existen varios índices disponibles, es fundamental identificar aquellos que presentan mayor precisión y fiabilidad en las diferentes etapas de la atención. Además, existe una laguna en los estudios dirigidos a evaluar la capacidad predictiva de los índices de gravedad en relación con los ingresos en la UTI y en el hospital. Ante este escenario, el objetivo de este estudio es comparar el rendimiento de los índices de trauma (ISS, NISS, REMS y mREMS) en la predicción de estos resultados.

## MÉTODO

### Diseño del Estudio

Estudio de cohorte retrospectivo, redactado de acuerdo con las recomendaciones del *STrengthening the Reporting of OBservational studies in Epidemiology* (STROBE)^(18)^, realizado mediante el análisis de historias clínicas electrónicas de pacientes traumatizados ingresados en el servicio de urgencias de un hospital privado de la ciudad de São Paulo. Se recopilaron datos sociodemográficos, así como datos relacionados con el traumatismo, la atención prehospitalaria y la atención hospitalaria.

### Lugar del Estudio

El estudio se llevó a cabo en un hospital privado, situado en la zona sur de la ciudad de São Paulo, caracterizado por su gran tamaño, con más de 700 camas activas. El servicio de urgencias para adultos de este hospital cuenta con 34 camillas y 31 sillones para la atención y el cuidado de personas en situación de urgencia o emergencia que, dependiendo de su estado clínico, son derivadas a unidades específicas de tratamiento. La institución está considerada un centro de traumatología de tipo II, de referencia para la atención de pacientes traumatizados que tienen acceso al sistema de salud complementario o condiciones socioeconómicas que les permiten sufragar los costes de la asistencia necesaria.

El hospital cuenta con un equipo de atención traumatológica compuesto por un enfermero, un cirujano general y un traumatólogo, que es activado por el equipo de urgencias en función de criterios fisiológicos, anatómicos y del mecanismo de la lesión.

El hospital cuenta con unidades de UTI para adultos distribuidas en dos plantas, con un total de 54 camas activas. Cada cama está equipada con un monitor multiparamétrico continuo y una estructura para soporte ventilatorio, administración de fármacos vasoactivos, sedación y realización de procedimientos de alta complejidad. Estas unidades tienen un perfil asistencial general y los pacientes traumatizados son ingresados en cualquiera de las camas disponibles.

### Muestra y Criterios de Selección

La muestra de este estudio estuvo constituida por pacientes traumatizados ingresados en el servicio de urgencias para adultos entre el 1 de enero de 2020 y el 1 de enero de 2022, según la información proporcionada por el Servicio de Archivo Médico y Estadístico del hospital local donde se realizó el estudio.

Los participantes en el estudio cumplieron los siguientes criterios de inclusión: individuos con traumatismos contusos, penetrantes o mixtos; edad ≥18 años; ingresados en la institución hasta 24 horas después del evento traumático. Se excluyeron los individuos ingresados en parada cardiorrespiratoria, los pacientes con quemaduras y los trasladados desde otras instituciones, ya que los tratamientos previos al ingreso en el lugar del estudio pueden alterar las variables fisiológicas utilizadas para el cálculo de los índices de gravedad.

### Recolección de Datos

La recolección de datos se realizó tras la aprobación del proyecto de investigación por parte del Comité de Ética en Investigación y la Coordinación del Servicio de Urgencias de la institución. Tras obtener las autorizaciones institucionales, se solicitó al Servicio de Archivo Médico y Estadístico que enviara la lista con las direcciones electrónicas de todas las personas atendidas en el servicio de urgencias como consecuencia de accidentes o actos violentos durante el periodo de estudio.

En la lista proporcionada por este servicio, se identificaron, en una hoja de cálculo en formato Microsoft Excel, con acceso restringido a través de la red interna y el correo electrónico corporativo, los números de los historiales y las direcciones electrónicas de los pacientes ingresados por causas externas en el sector de urgencias durante el período establecido en la investigación.

A través de esta lista, se contactó a los sujetos elegibles por correo electrónico institucional, con el objetivo de invitarlos a participar en la investigación. El correo electrónico enviado contenía, en anexo, el instrumento de recolección de datos y el enlace para acceder al Término de Consentimiento Libre y Esclarecido (TCLE). En ausencia de respuestas a este enfoque, se realizaron nuevos intentos de contacto y se enviaron hasta cinco correos electrónicos con este contenido.

Los datos se recopilaron en el período comprendido entre julio de 2023 y julio de 2024, mediante consulta del historial clínico electrónico, tras la firma del TCLE. Las variables dependientes fueron la hospitalización y el ingreso en la UTI. Las variables independientes fueron los índices de gravedad: REMS, mREMS, ISS y NISS.

El REMS es un indicador fisiológico de gravedad que se compone de la Escala de Coma de Glasgow (ECGl), la frecuencia cardíaca (FC), la presión arterial media (PAM), la frecuencia respiratoria (FR), la saturación de oxígeno (SatO_2_) y la edad. Según los valores observados al ingresar en el servicio de urgencias, estas variables reciben una puntuación de cero a cuatro puntos, excepto la edad, que varía de cero a seis^(9)^. El REMS se desarrolló para aplicarse en pacientes no quirúrgicos ingresados en urgencias; sin embargo, se ha demostrado su aplicabilidad en pacientes traumatizados^(19)^.

Con el objetivo de mejorar su capacidad predictiva de los resultados en la población traumatizada, en 2017 se desarrolló el mREMS^(10)^. En este instrumento, se modificó la ponderación de las variables edad y ECGl, y la PAM se sustituyó por la presión arterial sistólica (PAS). Para el mREMS, la puntuación de la ECGl varía de 0 a 6 y los demás parámetros se puntúan de 0 a 4^(10,12)^. La suma de la puntuación obtenida en las variables del REMS y del mREMS da como resultado las puntuaciones de los índices, que varían de cero a 26, siendo las puntuaciones más altas las que indican mayor gravedad.

El cálculo del ISS^(11)^ y del NISS^(12)^ sigue las directrices de la Escala Abreviada de Lesiones (*Abbreviated Injury Scale* AIS)^(20)^, un manual que presenta una lista con la descripción de numerosas lesiones, utilizada para identificar cada lesión del paciente y su gravedad. Esta lista proporciona, para cada descripción de lesión, un identificador compuesto por siete números, siendo el último dígito el valor que se refiere a la gravedad de la lesión. El ISS y el NISS utilizan en su cálculo la gravedad de las lesiones establecida por el AIS y, mediante métodos específicos de cálculo, estiman la gravedad global de un paciente^(12)^. La puntuación del ISS y del NISS varía de 1 a 75, y un AIS de 6 asigna automáticamente un ISS de 75, lo que sugiere un cuadro fatal.

Para caracterizar la muestra estudiada, se recopilaron variables sociodemográficas (sexo y edad) e información relacionada con el evento traumático (mecanismo del trauma; causa externa; atención prehospitalaria; tipo de soporte; y transporte prehospitalario). Desde el ingreso hospitalario, se analizaron parámetros clínicos y, durante el período de hospitalización, se consideraron los abordajes quirúrgicos, el destino tras la atención en urgencias y el tiempo de estancia hospitalaria.

Los datos se recopilaron mediante un instrumento de recolección de datos y se elaboró una ficha de registro de lesiones para calcular los índices de gravedad anatómica.

### Análisis y Tratamiento de los Datos

Toda la información recopilada se almacenó en una base de datos computarizada, protegida con contraseña, a fin de garantizar la seguridad de la información. Además, todos los datos susceptibles de identificación fueron codificados y anonimizados. Se utilizó el *software Jamovi*® versión 2.3.26.0 para los análisis estadísticos.

Las causas externas identificadas en los historiales de los participantes en este estudio se clasificaron de acuerdo con el capítulo XX Causas externas de morbilidad y mortalidad (V01-Y98) de la Clasificación Internacional de Enfermedades, 10.^a^ revisión (CID 10)^(21)^. En los casos en que no se pudo especificar el traumatismo en este capítulo, se utilizó el código CIE T14.9, correspondiente a «traumatismo no especificado», perteneciente al capítulo XIX de dicha clasificación.

El REMS y el mREMS se calcularon a partir de los datos fisiológicos registrados en la atención inicial de los pacientes ingresados en el servicio de urgencias. Las puntuaciones de estos índices se establecieron sumando la puntuación asignada a las variables fisiológicas que componen los instrumentos^(9,10)^.

Para calcular el ISS, se sumaron los cuadrados de las puntuaciones AIS más altas de las tres regiones corporales más afectadas por el traumatismo^(11)^. Para determinar el ISS, se identificaron las lesiones más graves en cada una de las regiones afectadas y se seleccionaron las tres puntuaciones AIS más altas en regiones distintas. El cálculo se realizó sumando el cuadrado de estos tres valores: ISS = (AIS1)^2^ + (AIS2)^2^ + (AIS3)^2^. Para establecer el valor del NISS, se sumaron los cuadrados de las tres puntuaciones AIS más altas, independientemente de la región corporal afectada^(12)^.

Para establecer los valores del ISS y del NISS, se consideraron todas las lesiones de los pacientes. Cabe destacar que los índices anatómicos se calcularon por duplicado y de forma independiente. En caso de discrepancia entre los evaluadores, se consultó a un tercer miembro con amplia experiencia en la codificación de lesiones. Al final de esta etapa, todas las codificaciones y cálculos fueron revisados y validados por este tercer miembro.

Se realizó la prueba de *Shapiro-Wilk* para verificar la distribución de los datos y, dado que no presentaron una distribución normal, se aplicó la prueba de *Mann-Whitney* en las comparaciones de grupos de pacientes hospitalizados o no en unidades del hospital y en la UTI*.* Para evaluar la capacidad predictiva de los índices de traumatismo, se construyeron curvas *Receiver Operating Characteristic* (curvas ROC) para los desenlaces de interés del estudio (hospitalización y admisión en la UTI).

Se utilizó el intervalo de confianza del 95 % del área bajo la curva ROC (AUC/ROC) para inferir diferencias estadísticamente significativas entre los valores de AUC/ROC^(22)^. De forma complementaria, se calculó el índice de *Youden* para seleccionar el punto de corte y se obtuvieron las medidas de sensibilidad, especificidad, valor predictivo positivo (VPP), valor predictivo negativo (VPN) y precisión.

La categorización de los valores de las métricas AUC/ROC siguió el formato propuesto en la literatura científica: ≤ 0,6 indicación de comportamiento aleatorio; >0,6 y ≤0,7 capacidad de discriminación insuficiente; >0,7 y ≤0,8 se consideró aceptable; >0,8 y ≤0,9 buena capacidad de discriminación y; >0,9 excelente^(23)^. El nivel de significación adoptado en todos los análisis fue del 5 %.

### Aspectos Eticos

Para la realización de este estudio, se respetaron las Directrices y Normas Reguladoras de Investigaciones con Seres Humanos, emanadas de la Resolución N.º 466/12 del Consejo Nacional de Salud^(24)^. El proyecto fue sometido y aprobado por el Comité de Ética en Investigación (CEP), a través de la plataforma Brasil (dictamen n.º 6.107.403 – CAAE 68381923.4.0000.0071).

El TCLE se aplicó por medios electrónicos. Inicialmente, los investigadores se pusieron en contacto con los posibles participantes en el estudio por correo electrónico para invitarlos a participar. Junto con el texto de la invitación, se presentaron los archivos de recolección de datos en formato PDF y el enlace para acceder al TCLE, disponible a través de REDCap® (Vanderbilt, Nashville, TN).

Tras la firma, se envió al paciente y/o familiar una copia de los documentos para posibles consultas posteriores. En ese momento, los investigadores orientaron sobre la importancia de archivar la copia e informaron a los participantes que podían retirar su consentimiento en cualquier etapa del desarrollo del estudio. En caso de que esto ocurriera, se enviaría un correo electrónico confirmando la retirada de su información del estudio. El TCLE se aplicó preferentemente al paciente y, solo en casos de dificultad para comprender el término o en caso de fallecimiento, se aplicó al familiar.

## RESULTADOS

Durante el período del estudio, 264 pacientes fueron atendidos en el servicio de urgencias como consecuencia de accidentes o actos violentos. Tras la aplicación de los criterios de elegibilidad, se excluyó a 42 pacientes (15,90 %), 31 de ellos por ser menores de 18 años (11,74 %), nueve por haber sido trasladados desde otras instituciones (3,40 %) y dos por una causa externa específica (quemaduras) (0,75 %). De este modo, 222 pacientes con traumatismos cumplieron los criterios de elegibilidad (84,09 %).

A estos pacientes se les envió por correo electrónico el TCLE, solicitando su consentimiento para utilizar sus registros hospitalarios en la investigación. Sin embargo, 71 pacientes (31,98 %) no respondieron tras cinco intentos de contacto o se negaron a participar. Así, la muestra quedó compuesta por 151 participantes. Entre ellos, 75 pacientes (49,66%) tuvieron indicación de hospitalización y 14 (9,27%) fueron admitidos en la UTI.

La media de edad de los pacientes fue de 52,40 años, la mediana fue de 47 años, con valores mínimos y máximos de 19 y 100 años, respectivamente. La mayor frecuencia se observó en el sexo masculino (55 %), y el traumatismo contuso fue el principal mecanismo de las lesiones (95,40 %). Entre las causas externas, las caídas y los accidentes que involucraron a ciclistas traumatizados presentaron la mayor incidencia (43,71% y 16,56 %, respectivamente).

En cuanto a la atención prehospitalaria, el 57 % de los participantes no recibió este tipo de atención; los demás recibieron, con mayor frecuencia, atención básica (37,10 %) por parte de equipos terrestres (41,70 %), como se muestra en la Tabla 1.

**Tabla 1:** Datos relacionados con el trauma, la atención hospitalaria y los índices de gravedad del trauma de los participantes en la investigación (n = 151) – São Paulo, 2020–2022.

Variables relacionadas al trauma	
Mecanismo del trauma – n (%)	
Contundente	144 (95,40)
Penetrante	7 (4,60)
Causa externa / código CIE10 – n (%)	
Caídas / W00-W19	66 (43,71)
Traumatismo ciclista / V10-V19	25 (16,56)
Motociclista traumatizado / V20-V29	17 (11,26)
Ocupante de coche traumatizado / V40-V49	15 (9,93)
Peatón traumatizado / V01-V09	6 (3,97)
Exposición a fuerzas mecánicas inanimadas / W20-W49	6 (3,97)
Otros accidentes de transporte terrestre / V80-V89	4 (2,65)
Otros traumatismos* / V30-V39; V90-V94; X85-Y09; X58-X59; X60-X84	5 (3,31)
Traumatismo no especificado / T14.9	7 (4,64)
Tipo de asistencia prehospitalaria – n (%)	
Básico	56 (37,10)
Intermedio	5 (3,30)
Avanzado	4 (2,60)
Sin apoyo	86 (57,00)
Tipo de transporte prehospitalario – n (%)	
Aéreo	2 (1,30)
Terrestre	63 (41,70)
No aplicable	86 (57,00)
Constantes vitales al ingreso en Urgencias – mediana (C1–C3)	
Frecuencia cardiaca, latidos por minuto	77,00 (69,00–89,00)
Presión arterial sistólica, milímetros de mercurio	123,00 (105–142)
Presión arterial diastólica, milímetros de mercurio	85,00 (74,50–99,50)
Presión arterial media, milímetros de mercurio	97,70 (88,20–109,00)
Escala de Coma de Glasgow	15,00 (15,00–15,00)
Saturación periférica de oxígeno, %	98,00 (96,00–99,00)
Temperatura axilar, grados Celsius	36,1 (36,00–36,50)
Variables relacionadas con la atención hospitalaria	
Destino tras el tratamiento en urgencias – n (%)	
Alta hospitalaria	76 (50,33)
Centro quirúrgico	25 (16,56)
Observación en urgencias	1 (0,66)
Unidad de hospitalización	31 (20,53)
Unidad semi-intensiva	4 (2,65)
Unidad de Cuidados Intensivos	14 (9,27)
Abordaje quirúrgico – n (%)	
Sí	51 (33,80)
No	100 (66,20)
Traslado hospitalario – n (%)	
Sí	13 (8,60)
No	138 (91,40)
Resultado – n (%)	
Superviviente	150 (99,30)
Muerte	1 (0,70)
Duración de la estancia hospitalaria – en días	
Mediana (C1–C3)	1,00 (0,00–3,00)
Índice de gravedad del traumatismo, mediana (T1–T3)	
*Índice de gravedad de la lesión*(ISS)	3,50 (1,00–8,00)
*Nuevo índice de gravedad de la lesión*(NISS)	3,50 (1,00–9,00)
*Escore rápido de urgencias*(REMS)	2,00 (0,00–5,00)
*Escore rápido de urgencias modificado*(mREMS)	1,00 (0,00–3,00)

Datos expresados en número y porcentaje (%) o en mediana y cuartil 1 (25%), cuartil 1 y cuartil 3 (75%), C3. (*) Otros traumatismos: ocupante de un triciclo / V30-V39 (n = 1); accidentes de transporte por agua / V90-V94 (n = 1); exposición accidental a otros factores o factores no especificados / X58-X59 (n = 1); lesiones autoinfligidas / X60 X84 (n = 1); agresión / X85-Y09 (n = 1).

Se observó que el 2,71 % de los participantes presentaban algún tipo de alteración en los parámetros clínicos al ingresar en urgencias. Se indicó tratamiento quirúrgico a 51 pacientes (33,80 %) y 13 participantes en el estudio (8,60 %) fueron trasladados a otro hospital. La mediana de la estancia hospitalaria fue de 1,00 día (C1: 0,00 – C3: 3,00) y solo se registró una muerte hospitalaria. Los valores medianos de los índices de gravedad se describen en la Tabla 1.

Como se muestra en la Tabla 2, los pacientes que requirieron hospitalización tenían puntuaciones de gravedad del traumatismo más altas que los que no fueron hospitalizados. Hubo una diferencia estadísticamente significativa entre los grupos con y sin hospitalización en relación con los índices analizados. Al comparar los pacientes ingresados o no en la UCI, se observaron valores más elevados entre los ingresados en esta unidad. Hubo diferencia estadísticamente significativa en los valores de ISS (p < 0,001), NISS (p < 0,001) y REMS (p = 0,027); sin embargo, no hubo diferencia estadísticamente significativa en los valores de mREMS (p = 0,103).

**Tabla 2:** Comparación de los índices de gravedad de los pacientes traumatizados con y sin hospitalización e ingreso en UCI – São Paulo, 2020–2022.

Índice de gravedad del trauma, mediana (C1–C3)	Hospitalización		p^M^
	Sí (n = 75)	No (n = 76)	
Índice de gravedad de la lesión(ISS)	6,00 (4,00–13,00)	1,00 (1,00–2,00)	<0,001
*Nueva puntuación de gravedad de la lesión* (NISS)	6,00 (4,00–15,00)	1,00 (1,00–2,00)	<0,001
*Puntuación rápida en medicina de urgencias* (REMS)	2,00 (0,00–6,00)	0,00 (0,00–4,00)	0,007
*Puntuación rápida de medicina de urgencias modificada* (mREMS)	2,00 (1,00–4,00)	0,00 (0,00–3,00)	0,002
**Índice de gravedad del traumatismo, mediana (C1–C3)**	**Ingreso en UCI**		**p^M^**
	**Sí (n = 14)**	**No (n = 137)**	
*Puntuación de gravedad de la lesión* (ISS)	10,00 (8,25–13,80)	2,00 (1,00–5,00)	<0,001
*Nueva puntuación de gravedad de la lesión* (NISS)	14,00 (9,00–17,00)	2,00 (1,00–6,00)	<0,001
*Puntuación rápida de urgencias* (REMS)	5,50 (1,00–7,00)	2,00 (1,00–4,00)	0,027
*Puntuación rápida de medicina de urgencias modificada* (mREMS)	2,00 (0,50–4,75)	1,00 (0,00–3,00)	0,103

Datos expresados como mediana y rango intercuartílico. ^M^,Prueba de *Mann-Whitney.*

El análisis de la capacidad predictiva de hospitalización mostró que el ISS y el NISS presentaron un buen rendimiento en la identificación del resultado (AUC/ROC: 0,84; IC95% = 0,78 – 0,90 y AUC/ROC: 0,85; IC95 % = 0,78 – 0,91, respectivamente). Sin embargo, el rendimiento del REMS y del mREMS se consideró insatisfactorio (AUC/ROC: 0,62; IC95% = 0,53 – 0,71 y AUC/ROC: 0,64; IC95 % = 0,55 – 0,73, respectivamente). Además, los IC95 % de los índices indicaron que el rendimiento del ISS y del NISS fue similar, ya que presentaron valores superpuestos, pero diferentes de los valores observados para el REMS y el mREMS, que también presentaron rendimientos similares entre sí. El área bajo la curva se ilustra en la Figura 1(A) y las demás métricas relacionadas con el rendimiento de los índices en relación con la predicción de hospitalización están disponibles en la Tabla 3.

**Figura 1:** (A). AUC/ROC de la capacidad predictiva de los índices de traumatismo en la predicción de la hospitalización; (B). AUC/ROC de la capacidad predictiva de los índices de traumatismo en la predicción de la admisión en la UTI – São Paulo, 2020–2022.

AUC/ROC – *Area under curve/Receiver Operating Characteristic Curve*; ISS – *Injury Severity Score*; NISS – *New Injury Severity Score*; REMS – *Rapid Emergency Medicine Score*; mREMS – *modified Rapid Emergency Medicine Score*.

**Tabla 3:** Métricas ROC de los índices de gravedad en relación a los resultados de ingreso hospitalario y en UCI – São Paulo, 2020–2022.

Métrica ROC para hospitalización	Índices de gravedad			
	ISS	NISS	REMS	mREMS
AUC/ROC (IC 95%)	0,84 (0,78–0,90)	0,85 (0,79–0,91)	0,62 (0,53–0,71)	0,64 (0,55–0,73)
Punto límite	3,5	2,5	1	0,5
Sensibilidad	0,78	0,83	0,70	0,76
Especificidad	0,78	0,75	0,51	0,52
Valor predictivo positivo	0,78	0,77	0,57	0,60
Valor predictivo negativo	0,78	0,82	0,65	0,69
Precisión	0,78	0,79	0,60	0,64
**Métricas ROC para ingresos en UCI**	**ISS**	**NISS**	**REMS**	**mREMS**
AUC/ROC (IC 95%)	0,85 (0,77–0,93)	0,85 (0,76–0,93)	0,67 (0,50–0,84)	0,62 (0,45–0,79)
Punto límite	4,5	5,5	3,5	1,5
Sensibilidad	0,92	0,92	0,71	0,71
Especificidad	0,70	0,71	0,68	0,58
Valor predictivo positivo	0,25	0,25	0,19	0,15
Valor predictivo negativo	0,98	0,98	0,95	0,95
Precisión	0,72	0,73	0,68	0,60

AUC/ROC Área bajo la *curva/curva característica operativa* del receptor; IC 95%, intervalo de confianza del 95%; ISS: *Injury Severity Score*; NISS: *New Injury Severity Score*; REMS: *Rapid Emergency Medicine Score*; mREMS: *modified Rapid Emergency Medicine Score.* Muestra (n = 151); Estancia hospitalaria (n = 75), Estancia en cuidados intensivos (n = 14).

De manera similar a lo observado en relación con la hospitalización, el ISS y el NISS presentaron un buen rendimiento en la discriminación del desenlace de ingreso en la UTI (AUC/ROC: 0,85, para ambos; IC95 % = 0,77 – 0,93 y 0,76 – 0,93, respectivamente). Sin embargo, el rendimiento del REMS y del mREMS fue insuficiente (AUC: 0,67; IC95 % = 0,50 – 0,84 y AUC/ROC: 0,62; IC95 % = 0,45 – 0,79, respectivamente), pudiendo clasificarse como aleatorio, según los valores estimados en el IC del 95 % del AUC/ROC. La representación del AUC se muestra en la Figura 1(B), y los datos complementarios de las métricas del AUC/ROC se muestran en la Tabla 3.

## DISCUSIÓN

La incorporación de índices con buen rendimiento para predecir la trayectoria hospitalaria y el pronóstico de los pacientes en la práctica clínica y en la gestión hospitalaria puede ser una estrategia valiosa para optimizar la asignación de recursos humanos y materiales. Además, su uso puede contribuir a un enfoque más estructurado y personalizado en la atención a pacientes con traumatismos, ayudando en la toma de decisiones y mejorando la calidad de la asistencia prestada. Desde esta perspectiva, se observó que el ISS y el NISS presentaron una buena capacidad predictiva de hospitalización y de ingreso en la UTI tras el ingreso en el servicio de urgencias, mientras que el REMS y el mREMS tuvieron un rendimiento insatisfactorio (AUC /ROC ≤ 0,7) o incluso aleatorio, cuando se consideró el IC95% observado (valores ≤ 0,6).

Se observó que aproximadamente la mitad de los individuos admitidos en el servicio de urgencias del lugar del estudio fueron derivados a unidades de hospitalización del hospital y, como era de esperar, los valores medianos de los índices de gravedad analizados fueron superiores (p ≤ 0,05) entre los pacientes hospitalizados, en comparación con los pacientes que recibieron el alta hospitalaria. Sin embargo, la puntuación media del ISS y del NISS entre los pacientes hospitalizados no alcanzó valores ≥ 16, indicativos de traumatismo grave^(25,26)^, y las puntuaciones del REMS y del mREMS fueron <6, lo que apunta a un bajo riesgo de mortalidad hospitalaria de los pacientes^(6,10,13)^.

En las decisiones relacionadas con la atención de pacientes traumatizados, la edad y el tipo de lesión diagnosticada son factores relevantes que pueden indicar la necesidad de hospitalización, independientemente de la gravedad del traumatismo y de las alteraciones fisiológicas observadas al ingreso. La literatura ha destacado la relación entre la edad, el traumatismo craneoencefálico y los peores pronósticos^(27)^; en consecuencia, en la práctica clínica se observa la adopción de conductas diferenciadas para los pacientes de edad avanzada y con este tipo de lesión.

En cuanto a la capacidad predictiva de los índices de gravedad en relación con el resultado de la hospitalización de pacientes con traumatismos, se observó que el ISS y el NISS presentaron un rendimiento similar, como lo demuestran los valores del IC95 % superpuestos. Las métricas para los puntos de corte establecidos —3,5 para el ISS y 2,5 para el NISS— también fueron similares, lo que no indica diferencias en el rendimiento entre los dos índices. Basándose en la fórmula de cálculo de estos índices (suma de los cuadrados de tres puntuaciones AIS) y en los puntos de corte establecidos para la hospitalización, se puede deducir que la presencia exclusiva de lesiones leves (AIS 1) discriminó a los pacientes que fueron dados de alta tras recibir atención en urgencias, teniendo en cuenta que esta escala clasifica las lesiones leves con una puntuación de uno, las moderadas con dos, las graves con tres, las muy graves con cuatro, las críticas con cinco y las de máxima gravedad con seis^(20)^.

El REMS y el mREMS presentaron un peor rendimiento en la discriminación de los casos que requirieron hospitalización, con un rendimiento insatisfactorio en todas las métricas analizadas, excepto en la sensibilidad. Según los IC95 % del AUC/ROC, se observó un rendimiento equivalente entre el REMS y el mREMS, pero distinto del ISS y el NISS.

En este estudio, la tasa de ingreso en la UTI fue inferior a la de otros estudios brasileños, que informaron un 11,50 %, un 24,80 % y un 81,90 % de ingresos en la UTI entre los pacientes con traumatismos^(15,16,28)^. Los valores medianos de los índices fueron más elevados entre los pacientes ingresados en la UTI, con una diferencia estadísticamente significativa solo para el ISS, el NISS y el REMS (p < 0,001 para el ISS y el NISS; p = 0,027 para el REMS). En un estudio realizado en un hospital de salud complementaria^(15)^, se observó una diferencia estadísticamente significativa en los valores de los tres índices (ISS, NISS y mREMS; p < 0,001).

También se evaluó la capacidad predictiva de los índices de gravedad en relación con el ingreso en la UTI, destacando el rendimiento del ISS y del NISS, ambos con AUC/ROC de 0,85. Considerando puntos de corte de 4,5 (ISS) y 5,5 (NISS), se observó un rendimiento similar entre los dos índices, con alta sensibilidad (0,92), lo que indica que el 92 % de los pacientes ingresados en la UTI presentaron valores por encima de los respectivos puntos de corte. Sin embargo, el VPP fue de 0,25 para ambos índices (ISS y NISS), lo que refleja el elevado número de falsos positivos, es decir, pacientes con valores por encima del punto de corte que no fueron ingresados en la UTI (n = 39 para el ISS y n = 38 para el NISS). Solo un paciente con valores inferiores a los puntos de corte fue ingresado en la UTI.

Por otro lado, el REMS y el mREMS mostraron un rendimiento insuficiente (AUC/ROC de 0,67 y 0,62, respectivamente), siendo superados por el ISS y el NISS en todos los análisis. Corroborando estos hallazgos, un estudio previo indicó que el ISS y el NISS tuvieron un mejor rendimiento en la predicción de la admisión en la UTI (AUC/ROC: 0,91; IC95 % = 0,88–0,94) en comparación con otros índices, como el Revised Trauma Score (RTS), el New Trauma Score (NTS), el mREMS, Trauma and Injury Severity Score (TRISS) y New Trauma and Injury Severity Score (NTRISS). Al igual que en el presente estudio, el mREMS presentó un rendimiento insuficiente (AUC/ROC: 0,64; IC95 % = 0,58 – 0,70) y bajos valores predictivos positivos, 0,44 (ISS), 0,52 (NISS) y 0,21 (mREMS)^(15)^.

En general, los resultados de este estudio sugieren que el REMS y el mREMS tienen un papel limitado en las decisiones sobre la trayectoria de los pacientes tras el ingreso en el servicio de urgencias (alta, ingreso hospitalario e ingreso en la UTI), mientras que el ISS y el NISS demuestran su potencial aplicación clínica en este contexto. El bajo VPP de ambos índices para la admisión en la UTI alerta sobre la importancia de las condiciones estructurales que influyen en esta decisión, tales como la capacidad de las unidades para monitorizar a los pacientes y la disponibilidad de camas en la UTI.

En la literatura, los índices de gravedad del trauma basados en parámetros fisiológicos han sido superados por los índices anatómicos en la predicción de diferentes resultados^(29)^. Índices como el ISS y el NISS son estables y presentan una puntuación única, ya que en su cálculo se tienen en cuenta exclusivamente las lesiones diagnosticadas en el traumatismo. Por otro lado, índices como el REMS y el mREMS, que utilizan parámetros fisiológicos, dependen de las intervenciones clínicas y de la evolución postraumática y varían en función de la atención prestada a los pacientes y de las características de la población estudiada. Los procedimientos iniciados en la atención prehospitalaria pueden estabilizar las condiciones circulatorias y ventilatorias, atenuando la gravedad expresada por índices basados en signos vitales iniciales medidos en el servicio de urgencias.

A pesar de estos aspectos, los parámetros fisiológicos son esenciales, sobre todo en la atención inicial, cuando las lesiones aún no han sido diagnosticadas completamente. En este caso, la aplicación inmediata de índices como el REMS puede ayudar a identificar precozmente el riesgo elevado de mortalidad.

Entre las limitaciones de este estudio, cabe destacar el tamaño de la muestra y el hecho de que la población pertenece a un centro de traumatología privado, con infraestructura avanzada y un perfil de pacientes diferenciado, lo que limita la generalización de los hallazgos al sistema público de salud. La muestra incluyó predominantemente pacientes con baja gravedad, muchos de los cuales fueron dados de alta directamente desde la sala de emergencias.

Además, se trata de un estudio retrospectivo basado en registros iniciales del departamento de emergencias, sin posibilidad de garantizar la estandarización temporal de la recolección de parámetros fisiológicos, lo que puede afectar la precisión de los índices fisiológicos. Además, los índices anatómicos (ISS y NISS) requieren un diagnóstico completo de las lesiones, lo que a menudo se produce después de las intervenciones, lo que limita su aplicación inmediata en la atención inicial. Sin embargo, este estudio se llevó a cabo en un hospital reconocido como centro de traumatología, con un equipo multidisciplinar y una infraestructura adecuada, que cumple los criterios técnicos exigidos para esta clasificación. Aunque se trata de una sola institución, el hospital atiende a pacientes con diferentes condiciones de acceso a la salud complementaria, abarcando diversos individuos y planes de salud, lo que amplía la diversidad de la población estudiada.

Los estudios en este contexto son fundamentales para describir una realidad poco explorada en la literatura científica nacional e internacional, permitiendo comprender la dinámica de la atención al trauma en la salud complementaria. Esto contribuye a la toma de decisiones basadas en la evidencia, la cualificación de la atención y una integración más eficiente con la red pública, sobre todo tras la implementación del Programa de Atención al Trauma en la Salud Suplementar. Esta integración es estratégica para reducir la sobrecarga de los hospitales públicos, anteriormente responsables de la atención de todos los casos de trauma.

## CONCLUSIÓN

Los índices anatómicos mostraron un buen rendimiento para discriminar a los individuos que necesitaron hospitalización y UTI. Por el contrario, los índices fisiológicos mostraron un rendimiento insuficiente para estos resultados. Se observó que el ISS y el NISS tuvieron un rendimiento similar entre sí, al igual que el REMS y el mREMS.

Los resultados indicaron que el ISS y el NISS pueden aplicarse clínicamente en la identificación de pacientes que necesitan hospitalización y UTI, mientras que el uso del REMS y el mREMS no se recomienda para este fin, según los hallazgos de este estudio.

Al igual que para otros profesionales de la salud, para el personal de enfermería, la información sobre el rendimiento de los índices de gravedad del trauma aporta valiosos datos para el uso de estos instrumentos en la práctica clínica y la gestión hospitalaria, garantizando la seguridad del paciente y mejores resultados en la atención a pacientes con trauma.

## Data Availability

El conjunto completo de datos que respalda los hallazgos de este estudio está disponible, previa solicitud razonable, a través de la autora de correspondencia (Lillian Caroline Fernandes).
